# Conservation of OFD1 Protein Motifs: Implications for Discovery of Novel Interactors and the OFD1 Function

**DOI:** 10.3390/ijms26031167

**Published:** 2025-01-29

**Authors:** Przemysław Jagodzik, Ewa Zietkiewicz, Zuzanna Bukowy-Bieryllo

**Affiliations:** Institute of Human Genetics Polish Academy of Sciences, Strzeszynska 32, 60-479 Poznan, Poland; przemyslaw.jagodzik@igcz.poznan.pl (P.J.); ewa.zietkiewicz@igcz.poznan.pl (E.Z.)

**Keywords:** oral-facial-digital protein 1, short linear motifs, evolutionary conservation, protein–protein interactions

## Abstract

OFD1 is a protein involved in many cellular processes, including cilia biogenesis, mitotic spindle assembly, translation, autophagy and the repair of double-strand DNA breaks. Despite many potential interactors identified in high-throughput studies, only a few have been directly confirmed with their binding sites identified. We performed an analysis of the evolutionary conservation of the OFD1 sequence in three clades: 80 Tetrapoda, 144 Vertebrata or 26 Animalia species, and identified 59 protein-binding motifs localized in the OFD1 regions conserved in various clades. Our results indicate that OFD1 contains 14 potential post-translational modification (PTM) sites targeted by at least eight protein kinases, seven motifs bound by proteins recognizing phosphorylated aa residues and a binding site for phosphatase 2A. Moreover, OFD1 harbors both a motif that enables its phosphorylation by mitogen-activated protein kinases (MAPKs) and a specific docking site for these proteins. Generally, our results suggest that OFD1 forms a scaffold for interaction with many proteins and is tightly regulated by PTMs and ligands. Future research on OFD1 should focus on the regulation of OFD1 function and localization.

## 1. Introduction

OFD1 (previously known as CXorf5 or 71-7) is a protein encoded by *OFD1* (ENSG00000046651) localized on the X chromosome (Xp22.2). The canonical transcript, OFD1-201 (ENST00000340096.11, NM_003611.2), is expressed from 23 exons and yields a 1012 amino acid (aa) protein. The OFD1-201 isoform contains several important domains, some of which are missing from the shorter splice isoform. The full-length OFD1 protein is a part of the centrosome complex, composed of centrosomes (microtubule-based centrioles) embedded in the amorphous pericentriolar matrix [1,2] and surrounded by protein-rich granules known as centriolar satellites (CSs) [3,4]. OFD1 is also found in the ciliary basal body and transformed centriole, which forms the cilium during interphase [5]. Moreover, OFD1 is also found in the nucleus [6].

OFD1 has been shown to play an essential role in many cilia- and centrosome-related processes, such as the biogenesis of primary and motile cilia [7], cilia maintenance [8,9], and mitotic spindle assembly [10]. Growing evidence suggests that OFD1 may also be involved in other cellular processes, including centrosomal protein translation [11], selective protein autophagy [12,13], and the repair of double-strand DNA breaks in the nucleus [6,14]. Mutations in *OFD1* cause several disorders, which affect primary and/or motile cilia (ciliopathies), and therefore manifest in multiple tissues. The severity of OFD1-related ciliopathies largely depends on the size of protein truncation [15,16,17,18,19,20].

One of the ways to better understand OFD1 functions in cellular processes and associated pathologies requires deciphering its interactions with other proteins. Protein–protein interactions (PPIs) are fundamental to virtually all biological processes. They occur when two or more protein molecules form a complex through various biochemical mechanisms, e.g., electrostatic forces, hydrogen bonding, van der Waals interactions and/or hydrophobic effects [21]. Typically, the surfaces of interacting proteins complement each other, both in terms of their physical structure and the chemical properties of the aa residues involved [22,23].

**Figure 1:**
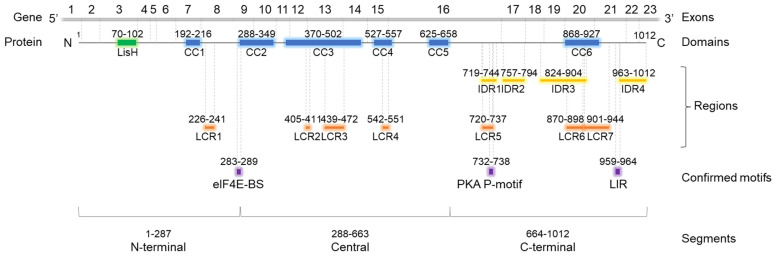
Domain architecture of OFD1 protein. Localization of LisH, CC, LCR according to SMART; localization of IDRs according to Uniprot database. Experimentally proven protein-binding motifs are according to [8,11,12]. The SMART database reports six CC domains in OFD1, spread throughout the protein length. Two are localized at the terminal parts of OFD1: CC1 close to the N-terminus, CC6 in the C-terminus [27]. Four CC domains (CC2–4), present in OFD1 central part, have been shown to mediate OFD1 dimerization [6]. SMART database reports seven LCRs in OFD1 identified using the SEG program [28]. One (LCR1) is localized in the N-terminal segment, between CC1 and CC2, three (LCR2–4) in the central part, and two (LCR5–6) in the C-terminal part of OFD1. LCR2–3, LCR4 and LCR6–7 are present within the CC3, CC4 and CC6 domains, respectively. In contrast to SMART, the Uniprot database does not report any LCRs. However, it does indicate the presence of four IDRs in the C-terminal part of the protein identified using the MobiDB-lite method [29]. Of the four IDRs, IDR3 partially overlaps with the CC6 domain. Discrepancies between the databases regarding the number and localization of CC domains are summarized in Figure S1. CC—coil-coiled domain; LisH—Lissencephaly type-1-like homology domain; IDR—intrinsically disordered region; LCR—low complexity region. Positions of three experimentally confirmed protein-binding motifs are shown: eIF4E-BS—eukaryotic initiation factor 4E binding site; PKA P-motif—protein kinase A phosphorylation motif; LIR—LC3-interacting region.

OFD1 protein contains several segments involved in protein binding (Figure 1), including two types of highly ordered domains. The LisH (Lissencephaly type-1-like homology) domain, consisting of 33 aa residues, forms two α-helices and binds microtubules (MTs) or cytoplasmic dynein heavy chains [24]. Coiled-coil (CC) domains, consisting of two or more α-helices wound around each other to form supercoils, contribute to protein stability, structure organization and protein interactions [25]. CC domains may interact with CC domains from the same protein (homo-oligomerization) or from another protein (hetero-oligomerization) [26].

Intrinsically disordered regions (IDRs) and low complexity regions (LCRs) are less defined. IDRs are protein segments that lack a stable three-dimensional structure and exist in a flexible, dynamic state. IDRs are biologically active and can interact with various proteins [30], acquiring a particular 3D structure upon partner binding. This structural flexibility allows IDRs to play crucial roles in diverse biological functions and cellular processes, such as signaling and regulation [31,32]; unfortunately, due to the dynamic character of these structures, predicting protein partners based solely on IDRs sequences is challenging, if not altogether impossible. Low complexity regions (LCRs), frequently found within CC domains or IDRs [33,34], are characterized by the reduced diversity of their aa composition. They may induce changes in the local structure of IDRs, influencing the mode of protein interaction [33].

Apart from the aforementioned domains and regions, a number of short linear motifs, of 3 to 15 adjacent aa residues, can be distinguished. Their physicochemical properties (e.g., identity, charge, polarity, size) enable them to fit the interaction surfaces of partner proteins, facilitating transient protein–protein interactions (PPIs) and post-translational modifications (PTMs). Motifs typically comprise a few highly conserved aa residues interspersed with less conserved positions, thus different instances of the motif within the protein can vary in sequence. Moreover, the flexibility in motif patterns enables variants of the same motif with different lengths to be detected at the same site, as long as the motif-specific aa residues fit the motif pattern criteria.

The cellular distribution of the OFD1 protein reflects its association with the centrosome/cilium complex and the MT cytoskeleton network. As a part of the centrosome/cilium complex, OFD1 participates in the MT cytoskeleton organization during mitosis and interphase and the process of cilia formation, maintenance and disassembly [7,8,10,35,36]. As a protein associated with the MT cytoskeleton network, OFD1 can affect the cytoskeleton structure and cell adhesion to the extracellular matrix [37]. OFD1 also participates in the regulation of protein degradation at the centrosome through ubiquitin-proteasome [38] and autophagy pathways [12]. In the nucleus, OFD1 interacts with the chromatin remodeling complex [6], takes part in the regulation of gene expression [39] and is involved in maintaining genomic stability [14].

All these functions require the interaction of OFD1 with other proteins. The recent high-throughput studies on cilia-interacting proteins have identified OFD1 in the center of a complex network of ciliary and centrosomal proteins, with over 300 interactors [40,41,42]. Previous studies have shown that LisH and the N-terminal/central CC domains may target OFD1 to CSs, while the central and C-terminal CC domains guide it to centrosomes [4]. However, the precise mechanisms and the participation of particular interactors with specific domains remain unclear. Also, the role of different OFD1 motifs in these interactions is only partially known. So far, protein interactions with three motifs in OFD1 have been experimentally confirmed: eukaryotic initiation factor 4E -binding site (eIF4E-BS), protein kinase A phosphorylation site (PKA P-motif) and LC3-interacting region (LIR) [8,11,12].

In this work, we aimed to shed light on OFD1 interaction networks by exploring conserved segments and motifs within the OFD1 sequence. Understanding PPIs involving OFD1 holds promise for deciphering the existing and possible novel OFD1 functions in cellular processes.

## 2. Results

### 2.1. Analysis of the OFD1 Protein Sequence Conservation

#### 2.1.1. OFD1 Sequence Analysis in 80 Species of Tetrapoda

**Figure 2:**
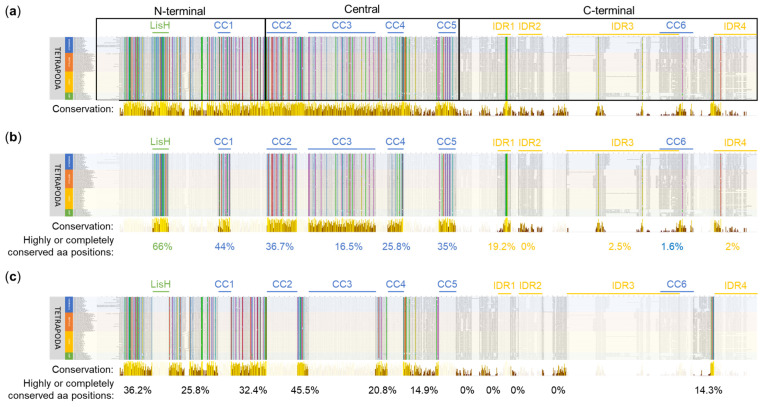
Multiple sequence alignment of OFD1 sequence from 80 species of Tetrapoda using Clustal. (**a**) Conservation along the whole protein length. N-terminal (aa positions 1–287), central (aa positions 288–663) and C-terminal (aa positions 664–1012) OFD1 segments are indicated. (**b**) Subset indicating conservation within the domains and IDRs. (**c**) Subset indicating conservation in the regions between the domains and IDRs. Bars below the alignment indicate the level of aa position conservation according to Jalview, and the numbers indicate percentage of highly or completely conserved aa positions within the indicated regions. Yellow color denotes more conserved, brown denotes less conserved positions. Animal names at the beginning of each line are highlighted depending on their phylogeny: mammals in blue, reptiles in red, birds in yellow and amphibians in green. The alignment was visualized using Jalview [45]. Identical aa residues or with similar properties at the same position are indicated by the Clustal X Default Coloring: blue—hydrophobic; red—positively charged; magenta—negatively charged; green—polar; pink—cysteines; orange—glycines; yellow—prolines; cyan—aromatic.

To reveal evolutionarily conserved regions in OFD1, we performed a multiple Clustal sequence alignment using OFD1 protein sequences from 80 Tetrapoda species (20 mammals, 24 reptiles, 27 birds and 9 amphibians). The alignment revealed positions, which were either highly conserved (aa residues had similar properties) or completely conserved (identical) across all the analyzed sequences. These positions were predominantly localized in the N-terminal and the central OFD1 regions, with significantly less presence in the C-terminal region (Figure 2a).

To verify the most conserved aa positions revealed in the alignment, a more detailed sequence logo analysis was performed [43,44]. The conservation score of aa positions in Tetrapoda OFD1 ranged from 0 to 5 bits per analyzed site, with the higher values corresponding to a higher conservation score (Figures S2 and S3).

LisH, the most conserved of all the OFD1 domains, had 66% (22/33) of its aa positions completely or highly conserved in Clustal alignment (Figure 2b). Among these, according to the sequence logo analysis, the most conserved aa positions (≥4 bits per site) were N75, H81, C85, Y87, Y89 and F94 (Figure S2).

The conservation of the CC domains was less pronounced: CC1, CC5, CC2, CC4 and CC3 contained 44% (11/25), 35% (11/34), 36.7% (22/60), 25.8% (8/31) and 16.5% (22/133) of highly conserved positions, respectively (Figure 2b). According to the sequence logo analysis, the most conserved aa positions, each with a conservation score of ≥4 bits, were as follows: Y203 and M215 in CC1; F310 and Q317 in CC2; N424, Y437, Q450, Q457 and Q483 in CC3; Q545 in CC4; and Y652 in CC5 (Figure S2). CC domains consisted primarily of hydrophilic residues, such as glutamic acid (E) (Figure S2, in blue).

Compared to CC domains, IDRs demonstrated much lower conservation scores in Clustal alignment, which is in agreement with their reported flexible structure [30]. A relatively high level of conservation (19.2% of completely or highly conserved aa positions) was found in IDR1, especially when compared to other IDRs (0–2.5%) (Figure 2b). According to the sequence logo analysis, one motif in this domain (RRLSSTP, positions 732–738) exhibited an intermediate conservation level (3 to 4 bits per site). An intermediate level of sequence conservation also characterized the SPI motif within the consensus sequence of IDR2 (not present in most mammals, including *H. sapiens*). Only in IDR4, W1012 exhibited a sequence conservation of ≥4 bits per site (Figure S2).

Many completely or highly conserved aa positions revealed by Clustal alignment were localized outside the known globular domains and IDRs. For example, 45% of the aa positions in the region between CC2 and CC3 (CC2–CC3) and 36.2% of aa positions in the region preceding the LisH domain (further referred to as 1–LisH) were highly or completely conserved (Figure 2c). According to the sequence logo analysis, these regions contained 19 aa positions with sequence conservation scores of ≥4 bits per site (Figure S3).

The majority of these conserved positions (13/19) were found in the N-terminal segment of OFD1, with four (Y24, Q25, F27 and Q39) present in the 1–LisH region, four (Y124, Q162, F183 and Y187) in the region between the LisH and CC1 (LisH–CC1) and five (F222, Q275, Y283, Q285 and Q287) in the region between CC1 and CC2 (CC1–CC2) (Figure S3).

In the central OFD1 segment, four highly conserved aa positions were localized between the CC2 and CC5 domains: two (Y353 and Y361) between CC2 and CC3 (CC2–CC3), one (Q622) between CC3 and CC4 (CC3–CC4) and one (Y558) between CC4 and CC5 (CC4–CC5) (Figure S3).

In the C-terminal part of OFD1, two conserved aa positions (Y961 and M962) were found between CC6 and IDR4 (CC6–IDR4). The presence of conserved aa positions may indicate the importance of these interdomain regions in the OFD1 function.

#### 2.1.2. OFD1 Sequence Analysis in 26 Species of Animalia

An alignment of OFD1 sequences from 26 representative species of the Animalia kingdom was performed to identify the most conserved aa positions among all classes of organisms expressing OFD1. Due to the relatively low number of the aligned sequences and their variation (resulting from large evolutionary distances), the maximal sequence conservation values in sequence logo analysis reached only 4 bits per site. The smaller number of sequences in this analysis reflected the limited availability of OFD1 sequences among some animal phyla.

**Figure 3:**
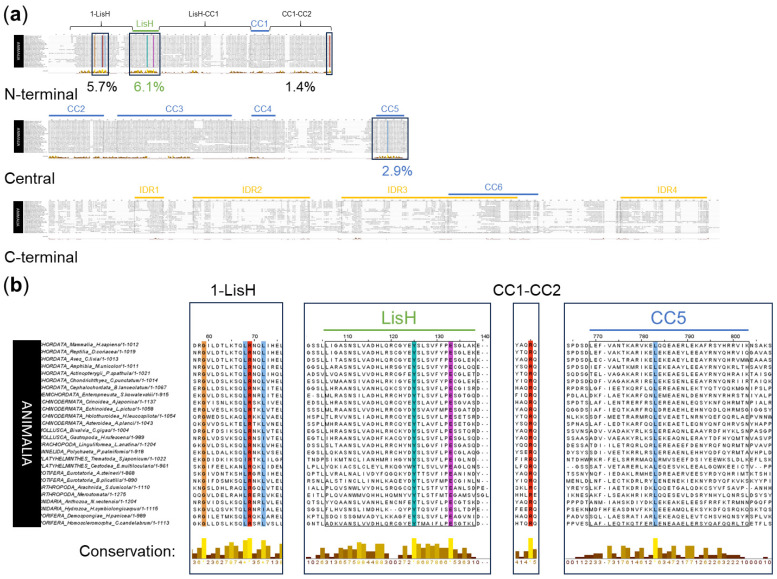
Multiple sequence alignment of OFD1 sequence from 26 species of Animalia using Clustal. (**a**) OFD1 protein divided into N-terminal, central and C-terminal regions. Boxes indicate areas containing completely or highly conserved aa residues, and numbers below the graph denote the percentage of highly or completely conserved aa positions according to Jalview. (**b**) These regions alone in magnification. Bars describe the level of conservation according to Jalview. Yellow color denotes more conserved and brown denotes less conserved positions. Coloring of the conserved aa residues as in Figure 2.

The Clustal alignment indicated eight aa positions in the N-terminal and the central OFD1 regions, which were conserved across the whole animal world (Figure 3a). Two completely conserved aa positions were in the LisH (Y89 and E97), and one was in the CC5 domain (L638); five completely or highly conserved aa positions were localized outside these domains (G31, L40, R41 and L44 in the 1-LisH, R286 in the CC1–CC2 region) (Figure 3b).

Sequence logo analysis identified 14 of the most conserved aa positions (the conservation score of 3–4 bits per site) (Figure S4). The four most conserved aa positions in the LisH domain in Animalia were H81, Y89, F94 and E97; three of them (except for E97) were the same as in Tetrapoda. Interestingly, multiple sequence alignment of 103 LisH domains from a variety of proteins from the Conserved Domain Database [46] (indicated with asterisk in Figure S4) did not list Y89 as conserved, indicating that the high conservation of this aa residue is specific to the LisH domain in OFD1.

Two highly conserved aa positions (D436 and Y437) were localized in CC3, and one (W1012) was in IDR4 (Figure S4). Outside the domains and IDRs, seven aa positions were highly conserved (G31, R41 in 1–LisH; Q162 in LisH–CC1; E264, R286, Q287 in CC1–-CC2; Y961 in CC6–IDR4) (Figure S4). The conservation scores for the remaining aa positions were <3 bits per site.

### 2.2. Identification of Short Linear Motifs in OFD1

**Figure 4:**
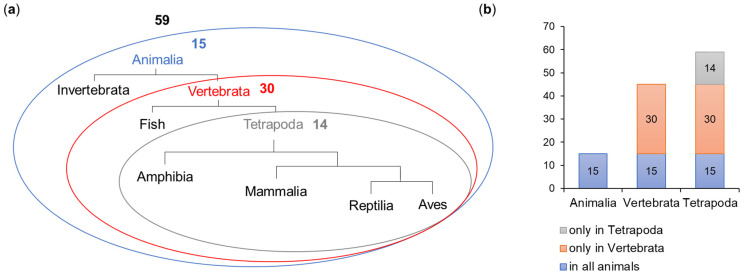
Evolution of the occurrence of motifs in OFD1. The number of motifs is shown as (**a**) simplified schematic phylogenetic tree and (**b**) diagram.

The ELM prediction tool detected 398 motifs, which represented 81 motif types (Table 1). Following the filtering procedure, 59 motifs representing 34 types (Table 1 and Table S1) were included in further analysis. A total of 15 motifs were shared across the whole animal kingdom, 30 were present in all Vertebrata and 14 were detected only in Tetrapoda (Figure 4).

The majority of 59 selected motifs were localized to the N-terminal and central part of OFD1, up to and including the CC5 domain (CC domains: 16 motifs; interdomain: 30 motifs; at the interdomain/domain borders: 7). Some motifs were also present in the C-terminus (3 motifs within IDRs, 3 motifs at the IDR borders) (Figures S6 and S7).

**Figure 5:**
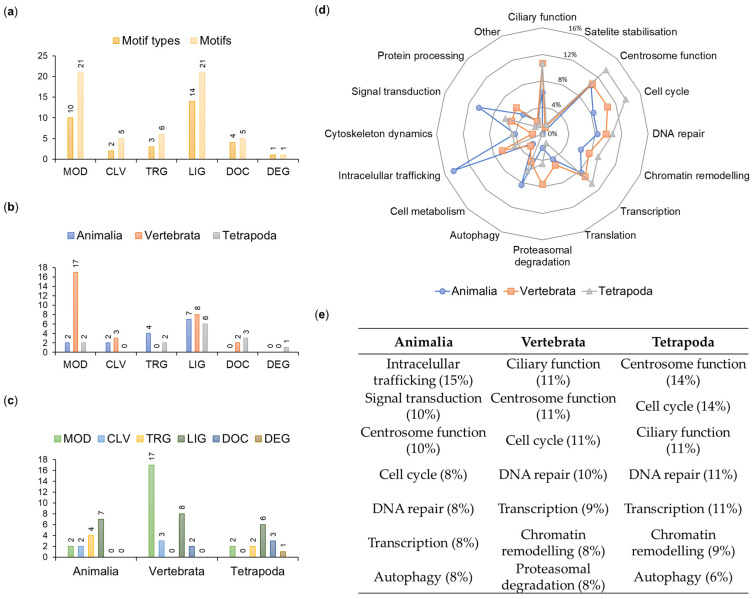
Characteristics of OFD1 motifs. (**a**) Distribution of motif types and motifs in the functional ELM classes. (**b**,**c**) Functional ELM classes in different clades sorted (**b**) by class and (**c**) by clade. (**d**) Proportion of clade-specific motifs involved in various functions. (**e**) Functional involvement of seven most frequent motifs in various clades. Percent values indicate proportion of the functional motifs within each clade.

A total of 59 motifs were distributed among six main functional classes listed in the ELM database: ligand binding (LIG—21 instances belonging to 14 types), post-translational modifications (MOD—21/10), protein targeting (TRG—6/3), protein-docking (DOC—5/4), protein cleavage (CLV—5/2) and protein degradation (DEG—1/1) (Figure 5a).

We assumed that the repeated occurrence of a given motif type within the protein sequence reflects the significance of the motif; therefore, when analyzing motif numbers across phyla or functional classes, we referred to the number of their instances (not types) within the OFD1 sequence (Figure 5).

#### 2.2.1. Evolutionary Conservation of the Identified Motifs

To gain insight into the evolutionary time when different OFD1 motifs were acquired, we analyzed the distribution of 59 motifs across the animal clades (Figure 5b,c). For example, MOD motifs were present in all clades but their number drastically increased in Vertebrata (from 2 to 17). DOC motifs appeared later in evolution (in Vertebrata and more in Tetrapoda), consistent with the conservation of the key aa residues positioned within these motifs. Interestingly, three aa positions identified earlier as the most conserved in the whole animal kingdom were found within the analyzed motifs (R41 in MOD_PIKK_1; D436 and Y437 in TRG_ENDOCYTIC_2; Y437 in LIG_LIR_Nem_3) (Figure S4).

The evolutionary age of the motifs was compared with the cellular function associated with interactors known to bind to specific motif types indicated by the ELM prediction tool. The three most frequent motifs present in all Animalia were mostly associated with intracellular trafficking, signal transduction and centrosome functions. Motifs common to Vertebrata and Tetrapoda were mostly associated with the ciliary and centrosome functions and the cell cycle (Figure 5d,e).

The literature search revealed that the indirect or direct interaction with the respective protein partner has been experimentally confirmed for only seven out of the fifty-nine motifs identified in our study (LIG_LIR_Gen_1 [12], LIG_eIF4E_1 [11,40], LIG_Dynein_DLC8_1 [40,47,48,49,50], MOD_Plk_1 [40,51], MOD_Plk_4 [52], MOD_PKA_1 [8] and DOC_CYCLIN_RxL_1 [40,53]) (see details in Tables S2–S4). The functional significance of the rest of the motifs needs to be experimentally validated.

#### 2.2.2. Functional Classification of the Identified Motifs

The most numerous among the 59 motifs identified in OFD1 (14 types, 21 in total) belonged to the functional class of LIG, associated with ligand binding. Five types of the LIG motifs were related to binding proteins (14-3-3 and proteins containing SH2, PTB or FHA domains) that recognize phosphorylated aa residues (SH2, PTB: phosphoTyr, 14-3-3: phosphoSer/phosphoThr, FHA: phosphoThr), indicating a possible role of OFD1 in signal transduction (Tables S1 and S2). The rest of the LIG motifs were related to binding various proteins (eIF4E, LIR, WIRS, WDR5, PCNA and DYNLL1—formerly known as DLC8) involved in other cellular processes, including protein translation [11], autophagy [12], actin cytoskeleton rearrangements [54], histone modifications [55], DNA replication and repair [56] and dynein transport [57] (Table S2). OFD1 interaction with eIF4E and proteins of the autophagy-related protein ATG8 family has already been proven [11,12].

**Figure 6:**
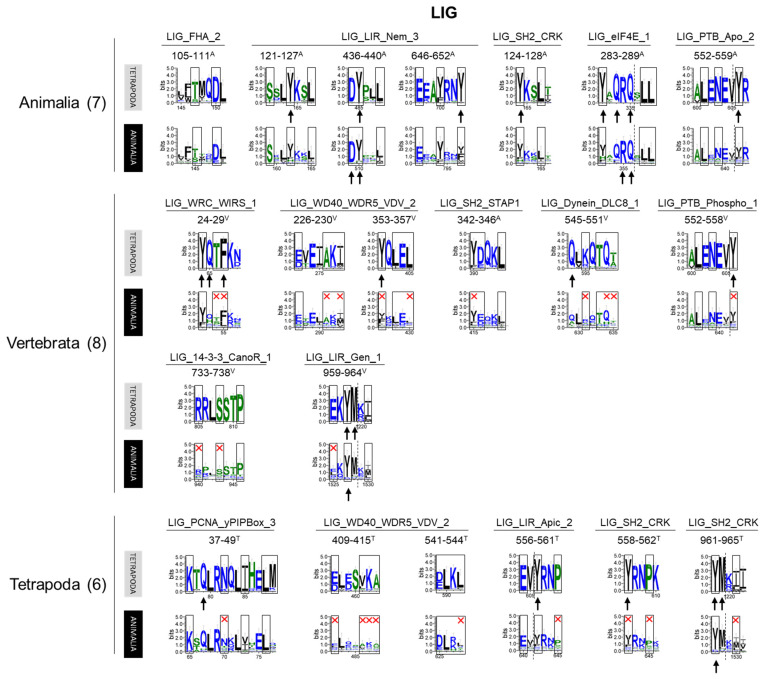
Sequences of the LIG motif sequences identified in human OFD1 and their conservation within different groups of animals. Motif patterns identified in human OFD1 were compared with the sequence logo graphs representing consensus sequence of Tetrapoda and Animalia alignments. Aa positions required by the motif pattern are boxed; red cross indicates aa residues not fitting the motif pattern. The most highly conserved aa positions in the protein alignments are indicated by an arrow. Color of the aa residues reflects its hydrophobicity (blue—hydrophilic; green—neutral; hydrophobic—black). Motifs are grouped according to the animal clades in which the motif was recognized as conserved. Similar analyses for remaining motif classes can be seen in Supplementary Materials.

Seven LIG motifs (belonging to five types) were present in the whole animal kingdom (Figure 6). The functions associated with these conserved motifs suggest the ancient nature of OFD1 interaction with translation, autophagy and signal transduction machinery (Table S2). The vertebrate-specific occurrence of LIG motifs associated with the binding of WIRS, DYNLL1 and WDR5 proteins (involved in actin cytoskeleton [54]; intracellular transport [57]; and histone modifications [55], respectively), indicated the acquisition of new binding partners during the evolution and expansion of OFD1’s functional repertoire. Additionally, novel instances of the LIG_LIR motifs (LIG_LIR_Gen_1, overlapping with LIG_LIR_Nem_3) that appeared in Vertebrata suggested the increased role of OFD1 in autophagy. LIG motifs, which only appeared in Tetrapoda included a novel autophagy-related LIG_LIR_Apic_2, PCNA-binding LIG_PCNA_yPIPBox_3, and two novel LIG_WD40_WDRS5_VDV_2 motifs. Their late appearance indicates the further evolution of the OFD1 complex interaction network.

A total of 21 various motifs (representing 10 motif types) belonged to the functional MOD class. The majority of the MOD motif types (8/10) are recognized by protein kinases, such as Protein Kinase A (PKA), Polo-like kinases 1 and 4 (PLK1, PLK4), Phosphoinositide 3-Kinase-related Kinase (PIKK) family, Casein Kinases 1 and 2 (CK1, CK2) and Proline-directed Kinases (PDK) (such as, e.g., mitogen-activated protein kinases, MAPKs). These proteins are known to be involved in the regulation of a range of cellular processes, including cell division [58,59,60,61], centriole duplication [59,60,61], cilia-related functions [62,63,64,65,66,67,68,69], cell cycle progression [59,60,70,71,72] and DNA checkpoint pathways [73,74,75,76] (Table S3). Two MOD motif types (seven motifs in total) were associated with the SUMO protein, known to affect protein localization, stability or important functions, e.g., cilia assembly and functioning [77,78] (Table S3).

The conservation of the key aa residues in MOD motifs recognized by PIKK and CK2 suggests that these two kinases may be the oldest OFD1 interactors (Figure S5). However, the majority of MOD motifs (17/21) binding other kinases and SUMO protein are conserved in Vertebrata, suggesting that OFD1 interactions with these proteins may have evolved at that time. The presence of two new instances of MOD motifs recognized by SUMO and PIKK kinases within the Tetrapoda-specific OFD1 sequence suggests the increased regulation of OFD1 function (Table S3).

Five motifs (four types) detected in OFD1 by the ELM tool belonged to the class of protein-docking (DOC) motifs (Table S4, Figure S5). Two of them are involved in the formation of complexes with MAPKs (DOC_MAPK_gen_1) involved in signal transduction and with cyclins and cyclin-dependent kinases (DOC_CYCLIN_Rxl_1) involved in cell cycle regulation. Another DOC motif is a docking site for protein phosphatase 2A (PP2A), the major cellular protein dephosphorylation enzyme, and for peptidyl-prolyl cis-trans isomerase NIMA-interacting 1 (PIN1) protein, which catalyzes proline cis to trans isomerization. Both phosphorylation and prolyl isomerization may change the protein conformation, influencing their PPIs and cellular functions. Motifs recognized by PIN1 overlap with the conserved aa positions identified in Vertebrata alignments, while the rest of aa positions in DOC motifs are conserved only in Tetrapoda.

An interesting and unexplored class of ELM tool-detected motifs was related to the processes of intracellular transport and targeting (TRG) (Table S5). TRG_ENDOCYTIC_2 motif is recognized by the µ subunit of AP-2 complex, the major protein involved in endocytosis. This motif, the most frequent in the TRG class (4/6), overlaps with the most conserved aa positions in OFD1. Tetrapoda-conserved TRG motifs included TRG_DiLeu_BaEn_1 and TRG_NES_CRM1_1, which are involved in vesicular trafficking and nuclear export, respectively (Figure S6).

The ELM tool detected five motifs (representing two types) in the protein cleavage (CLV) class, whose interacting proteins participate in intracellular trafficking (Table S5). The CLV_PCSK_SKI1 motif was detected at four OFD1 sites, two containing aa positions conserved in all animals, and two aa positions conserved in Vertebrata. Another CLV motif, CLV_NRD_NRD_1, was conserved in Vertebrata; its interactors, in addition to intracellular trafficking [79], have a role also in spermatogenesis [80].

The least represented class of ELM-detected motifs in OFD1 was related to protein degradation (DEG). The only motif in that class, DEG_APCC_DBOX_1, is recognized by Anaphase-Promoting Complex/Cyclosome (APC/C) proteins, which participate in protein degradation related to the cell cycle [81], autophagy [82] and centrosome functioning [83] (Table S5). This motif was found in the OFD1 sequence conserved only in Tetrapoda, suggesting that interaction with this protein might be acquired relatively late in the evolution.

Of note, some of the identified motifs are positioned adjacent to phosphorylation sites (MOD motifs), forming potential switches. The phosphorylation of the MOD motifs leads to either the activation or inhibition of protein–protein interactions in the neighboring motifs. Examples of such potential switch motifs include the following: LIG_PTB_Apo_2 [84] and LIG_PTB_Phospho_1 motifs [85] (both in CC4 region of OFD1), two DOC_WW_Pin1_4 motifs [86] (present in CC4–CC5 or IDR1) and four TRG_ENDOCYTIC_2 motifs [87] (present in 1–LisH, LisH-CC1, CC3 and CC6–IDR4 of OFD1) (Figure S7).

### 2.3. Summary of the Key Findings

The ELM-tool analysis of the evolutionary occurrence of protein motifs in human OFD1 revealed 59 motifs (representing 34 types) localized in the OFD1 regions conserved in various clades. The high number and early origin of the identified motifs are not surprising considering the involvement of OFD1 in a multitude of essential cellular processes and the predicted number of interactors [41].

## 3. Discussion

### 3.1. Confirmed and Novel OFD1 Interactors

Protein interactions with several of the ELM-detected motifs have already been experimentally confirmed [8,11,12], indicating that ELM predictions might indicate bona fide protein partners for OFD1.

The first of these OFD1 motifs (MOD_PKA_1 with Ser735, Tables S1 and S3) is related to binding PKA (Ser/Thr) kinase, which plays a crucial role in cellular processes such as metabolism, embryonic development, primary cilia biogenesis and resorption [8,88]. Recent studies have proven that PKA phosphorylates OFD1 at Ser735 (Figure 1—PKA P-motif), promoting its removal from centrosomes through the praja2-ubiquitin-proteasome system (UPS), an essential step in primary cilia formation [8].

LIG_eIF4E_1 is another experimentally confirmed OFD1 motif involved in PPI (Tables S1 and S2). It has been shown to mediate OFD1 interaction with eukaryotic translation initiation factor 4E (eIF4E), which is essential for the initiation of cap-dependent mRNA translation in the cytoplasm and in centrosomes. In addition, OFD1 has been shown to indirectly interact with other subunits of the translation initiation factors (eIF3B, eIF3G, eIF4G) (Figure 1—eIF4E-BS) [11].

The third experimentally proven OFD1 motif belongs to the LIR class and binds proteins from the ATG8 family (such as LLC3 and GABARAP) involved in the process of selective autophagy. OFD1 contains several LIR motifs spread in the protein center and C-terminus. Experiments confirmed that the C-terminal LIR motif (LIG_LIR_Gen_1, highly similar to LIG_LIR_Nem_3, see Table S1 and Figure 1—LIR motif) is essential for OFD1 involvement in autophagy. Mutations in the LIG_LIR_Gen_1 motif (EKYMKI to EKAMKI) completely abolish LLC3 and GABARAP binding and OFD1’s ability to induce ATG13 degradation [12,13].

We attempted to use the AlphaFold prediction tool to model the positions of the experimentally confirmed motifs in the OFD1’s 3D structure (Figure S8) and predict the 3D structure of OFD1 binding these experimentally confirmed partners (Figure S9). However, significant discrepancies between the AlphaFold predictions and experimental data rendered them unsuitable for analysis.

In addition to these confirmed interactions, our ELM data suggest a wide range of other proteins that bind to OFD1. Notably, interactions with these specific motifs are not yet verified. This can be caused first by the limitations of current 3D structure prediction tools but also by the short length of the linear motifs, which support transient PPIs, making it challenging to experimentally prove the interaction.

Other potential OFD1 interactors include a number of proteins, for whom predicted interactions with specific OFD1 motifs have not been experimentally confirmed. The first promising motif is the DYNLL1-binding motif (LIG_Dynein_DLC8_1). DYNLL1 plays a role as a cytoskeletal motor and is a hub protein interacting with proteins involved in many cellular processes, including apoptosis, nuclear transport, transcription and DNA repair [89,90]. The interaction between DYNLL1 and OFD1 has been previously identified in several proximity labeling and affinity capture-MS studies [40,47,48,49,50], but information about the DYNLL1-binding site was missing. Our work shows that the DYNLL1-binding motif (LIG_Dynein_DLC8_1) is present within the CC4 domain and is conserved in Vertebrata. It has been previously suggested that DYNLL1 binding to motifs present in CC domains may destroy existing interactions or inhibit other activities [90]. Thus, DYNLL1 binding to its motif might influence the composition of the OFD1-containing complexes, acting as a switch between, e.g., nuclear and cytoplasmic OFD1 functions; alternatively, it can inhibit other OFD1 processes. It is tempting to hypothesize that DYNLL1-OFD1 interaction supports OFD1 functions in centriolar satellites—structures present only in Vertebrata [91].

Next, the promising motifs are MOD_Plk_1, MOD_Plk_4 and MOD_CK2_1, recognized by PLK1, PLK4 and CK2 protein kinases, respectively. The direct interaction of PLK1 with OFD1 has been suggested by large-scale methods [40,51] and confirmed by a high-stringency Y2H system [51]. However, the binding sites are not established yet; perhaps, two MOD_Plk_1 motifs in the central part of OFD1 can play this role (Table S3, Figure S7b). PLK1 present at the pericentriolar matrix activates HDAC6 (histone deacetylase 6) to promote ciliary deacetylation and resorption [69]; it is also involved in mitosis regulation through its influence on centriole elongation, spindle assembly and mitosis-related DNA repair [61,76].

MOD_PLK_4 is a motif recognized by PLK4, another Ser/Thr protein kinase of the polo-like kinase family, essential for centriole duplication and control of centriole number [92]. So far, the close vicinity of PLK4 and OFD1 has been proven in proximity labeling assays [52]. The identification of a highly conserved PLK4-phosphorylation site at the central OFD1 part (Table S3, Figure S7b) provides further support for direct interaction between these proteins.

Other important motifs are MOD_CK2_1, binding the casein kinase 2 (CK2), a Ser/Thr protein kinase involved in the regulation of the cell cycle and its progression [59]. Our analysis has indicated three sites for CK2-driven phosphorylation in the OFD1 terminal part, with the most N-terminal motif conserved across the animal kingdom (Table S3, Figure S5). These observations support the earlier suggestion of direct interaction between these proteins, indicated by identifying OFD1 in close vicinity of CK2 [40]. This potential interaction is further supported by the presence of a docking site for cyclin N-terminal domain protein (DOC_CYCLIN_RxL_1), a regulatory subunit for CK2 [93]. The DOC_CYCLIN_RxL_1 motif is located in the CC3 domain of OFD1 and is conserved across Tetrapoda.

### 3.2. Conservation of Motif Classes

The selection of a range of potential OFD1 interactors, based on the possible or proven interactions with specific OFD1 motifs, indicates links to potential cellular processes in which OFD1 participates and suggests an evolutionary order in which these interactions might have evolved.

Our results indicate that LIG, TRG and CLV motifs in OFD1 appeared earliest in evolution and; their interacting proteins were primarily involved in intracellular protein trafficking, signal transduction and centrosome functioning (Figure 5d,e), functions fundamental for cell functioning in all organisms [94,95].

The majority of motifs conserved only in Vertebrata belonged to the MOD and LIG classes, and their interactors were predominantly related to cilia, centrosome functions and the cell cycle. The evolution of these motifs was possibly linked to processes involved in Vertebrata differentiation. An additional layer of regulatory control over OFD1 function in Vertebrata could have been exerted through the development of PTM signaling modules. Proteins interacting with motifs such as MOD_ProDKin_1 and LIG_14-3-3_CanoR_1 are implicated in CS stabilization [96]. The detection of the LIG motif binding protein DYNLL1 (LIG_Dynein_DLC8_1), involved in the transport of CSs between centrioles and cilia, alignment of the mitotic spindle and cell division [89,97], further supports the idea of the tighter regulation of centriole and ciliary processes in Vertebrata. In addition, a novel LIG motif associated with the LIG_LIR_Nem3 autophagy protein is also conserved in Vertebrata (Table S2). All this aligns with the reports of the vertebrate-specific appearance of CSs as structures that bring together ciliary/centriolar proteins and proteins involved in their synthesis and degradation [98].

Similarly to Vertebrata, OFD1 motifs conserved in Tetrapoda are associated with centrosomes, cilia and cell cycle regulation. The major motif classes are LIG and DOC, suggesting an additional level of fine-tuning of the OFD1-related processes, likely reflecting adaptations to terrestrial living conditions.

### 3.3. Motif Functions and Interactions

The most intriguing group of ELM-detected motifs in OFD1 was MOD, associated with PTMs such as SUMOylation and phosphorylation.

SUMO (Small Ubiquitin-like Modifier) proteins are known to participate in the regulation of the cell cycle, ciliogenesis, oo- and spermiogenesis, processes also related to OFD1. In most cases, SUMO modification regulates protein localization [99], e.g., the SUMOylation of centrin-2, the structural centrosome protein, constrains centrin-2 to the cytoplasm, preventing its shuttling to the nucleus [100]. SUMO can also modify cilia-associated proteins, such as the small ciliary GTPase, ARL13B; this modification is required for the ciliary localization of sensory receptors and polycystin-2 [101]. It is tempting to speculate that post-translational modifications by SUMO may similarly influence OFD1 localization.

Phosphorylation is a versatile mechanism, which drives signaling and regulates protein function [102]. It is orchestrated by protein kinases (“writers”), which add a phosphate group to the target proteins, phospho-binding proteins (“readers”), which recognize these modifications, and phosphatases (“erasers”), which remove them [103]. Beyond direct modification, phosphorylation may also allosterically change the structure of specific proteins, influencing their binding to other proteins [104].

Although it has been previously proven that OFD1 can be phosphorylated by Protein Kinase A (PKA) [8], our results suggested that as many as 14 potential PTM sites targeted by at least eight protein kinases are present in OFD1. This suggests that OFD1 regulation by phosphorylation is more complex and extensive than previously thought and potentially impacts a broader range of cellular processes. The presence of six various LIG motifs, which may interact with proteins recognizing phosphorylated Ser/Thr residues (“readers”), further suggests that changes in OFD1 phosphorylation may be detected by (and transferred to) other OFD1 interactors. This can also occur through motif switching, as exemplified by the close proximity of the MOD_PKA_1 and DOC_WW_Pin1_4 motifs within IDR1 or the overlap between MOD_PIKK_1, LIG_PTB_Apo_2 and LIG_PTB_Phospho_1 in CC4 of OFD1 [105] (Figure S7). The identification of an OFD1 motif involved in the binding of PP2A responsible for Ser/Thr dephosphorylation further underscores the possible dynamic regulation of OFD1 by phosphorylation.

Our findings also suggest that MAPKs, known for their roles in signal transduction, stress response, and cell cycle regulation [106], represent a particularly significant group of possible OFD1 interactors. OFD1 not only harbors a motif that enables phosphorylation by MAPKs (MOD_ProDKin_1) but also has a docking site specific for these proteins (DOC_MAPK_1). This dual relationship highlights the potential for a feedback loop, in which MAPKs both modify OFD1 and interact with it. As a part of the MAPK complex, OFD1 may play a critical role in MAPK-driven signaling pathways, potentially assisting MAPKs in transmitting phosphorylation signals through the formation of a scaffold organizing the MAPKs and its downstream targets. This suggests a broader functional involvement of OFD1 in fine-tuning the MAPK-mediated cellular responses.

Collectively, our findings highlight that OFD1 is tightly regulated by phosphorylation and that its modifications play a central role in modulating OFD1 interactions and functions within cellular signaling networks.

### 3.4. OFD1 Regulation by Intracellular Targeting and Protein Degradation

Our analysis for the first time revealed potential OFD1 interactors, which may be involved in the intracellular targeting of this protein. OFD1 contains four sites harboring the TRG_ENDOCYTIC_2 motif, recognized by the µ-subunit of the components of the Adaptor Protein (AP). AP are ancient protein complexes involved in clathrin-dependent transport [107,108,109]. The TRG_ENDOCYTIC_2 motif conservation across the whole animal kingdom (Table S1, Figure S6) confirms the significance of OFD1 transport within the cell. Interestingly, the epithelial-specific µ-subunit of AP complex (µ1B) is involved in the trafficking of proteins to the basolateral membrane, a process in which the role of OFD1 has been confirmed [109].

An additional motif related to intracellular protein sorting, TRG_DiLeu_BaEn_1, is localized in the LisH–CC1 region of OFD1. This motif is recognized by the sigma subunit of the AP-1-3 complexes, and, according to ELM, is involved in clathrin-mediated endocytosis or protein sorting of membrane proteins [105]. Interestingly, TRG_DiLeu_BaEn_1 motifs can work synergistically with the TRG_ENDOCYTIC_2 motifs [105]. This highlights the fact the importance of the correct protein localization to the various intracellular compartments.

The number of motifs recognized by proteins involved in proteolytic protein cleavage and degradation supports the notion that OFD1 activity and functions must be tightly controlled.

Interestingly, phosphorylation and proteasome degradation are known to regulate the number and protein content of CSs. It has been shown that in response to cellular stresses (UV, heat shock, transcription block), a centriole/CS protein CEP131 is phosphorylated by MK2 kinase (also known as Mitogen-Activated Protein Kinase-Activated Protein Kinase 2, MAPKAPK2), which induces CEP131 binding to 14-3-3 proteins and sequesters CEP131 in cytoplasm, preventing the formation of new CSs [110].

## 4. Materials and Methods

### 4.1. Quantity and Distribution of Coiled-Coil Domains

The number and localization of coiled-coil (CC) domains in the OFD1 structure were assessed using DeepCoil1 and DeepCoil2, neural network-based tools, with default settings [111]. DeepCoil is recommended because of its accuracy in detecting both canonical and non-canonical CCs [112]. Other bioinformatic tools for CC prediction were also used, such as PCOILS [113], Jpred4 [114] and Paircoil2 [115]—all with default settings. Paircoil2 [115] was additionally used with probability cutoff set to 0.1 (default probability cutoff is 0.5).

### 4.2. Protein Sequence Collection

OFD1 protein sequences from different species were retrieved from the National Center for Biotechnology Information database (NCBI, National Library of Medicine, Bethesda, MD, USA) using the Protein BLAST tool [116], with the human OFD1 sequence (UniProt: O75665) as a query.

Tetrapoda dataset of OFD1 sequences (80 entries) represented 20 species of mammals, 24 species of reptiles, 27 species of birds and 9 species of amphibians. Each species in the dataset represented a separate group or family. Phylogenetic tree of Tetrapoda OFD1 sequence (Figure S10) was consistent with the cladogram of mammals [117], reptiles [118,119,120,121], birds [122] and amphibians [123,124], indicating that the sequences were selected correctly.

The Vertebrata dataset consisted of Tetrapoda (80 species) supplemented with OFD1 sequences from 64 fish species encompassing Lungfish, Actinistia, Actinopterygii, Cartilaginous fishes and Jawless fishes. This dataset was used solely to assist in distinguishing Tetrapoda-specific motifs and was not intended for direct comparison between Tetrapoda and fish.

Animalia dataset consisted of OFD1 protein sequences from 26 species from different phyla: 7 representing Chordata (including 6 Vertebrata), 1 Hemichordata, 2 Mollusca, 1 Annelida, 1 Brachiopoda, 4 Echinodermata, 2 Platyhelminthes, 2 Arthropoda, 2 Rotifera, 2 Cnidaria and 2 Porifera. At least two species from separate classes for each major animal phylum were selected, except for Hemichordata, Annelida and Brachiopoda, where the number of OFD1 sequences was limited. Phylogenetic tree of OFD1 sequence in the Animalia dataset (Figure S11) was consistent with the phylogeny of animals [125], indicating that the sequences were selected correctly.

### 4.3. Multiple Sequence Alignment

Multiple sequence alignment of OFD1 sequence from 80 species of Tetrapoda, 144 species of Vertebrata (including 80 Tetrapoda) or 26 species of Animalia was performed using Clustal Omega (EBI) with default settings [126,127] and visualized using Jalview version: 2.11.4.1 [45].

### 4.4. Graphical Representation of Amino Acid Conservation

A more detailed analysis of the amino acid (aa) residue conservation in the groups was performed using sequence logo generator online tool, WebLogo, version: 3.7.12 [43,44] with default 3-color scheme, which reflects aa hydrophobicity. In this analysis, the conservation score expressed in bits takes into account the frequency of the aa residue at a particular position and the relative frequency of this residue within the whole set of analyzed protein sequences. If an aa residue is rare, a fully conserved position for that aa residue will have a higher information content (more bits); therefore, even for completely conserved positions, the number of bits can vary depending on the rarity of the aa residue in the protein [43,44]. The height of the symbols on the sequence logo graph reflects the conservation score.

### 4.5. Motif Searching and Filtering

Functional motifs were identified using the Eukaryotic Linear Motif (ELM) prediction tool [105,128] on the default settings, with the human OFD1 protein sequence (UniProt accession number: O75665) as a query. ELM is a comprehensive repository of experimentally validated, manually curated protein-binding motifs present in eukaryotic proteins and allows the identification of motifs in the submitted protein sequence [105,128].

ELM prediction tool by default removes motifs present in stable globular domains (LisH, CC), as they might not be accessible for surface interaction [128]. We accepted the exclusion of motifs present in the LisH domain. However, since the number and precise localization of CC domains varied depending on the CC domain prediction tool used (Figure S1) and previous studies have experimentally proved motif eIF4E-BS, which overlap with CC2 domain [11], we decided to keep motifs detected within CC domains. On the other hand, motifs in which motif pattern-specific aa residues were not conserved. Also, one motif without functional relevance to humans (TRG_Oom_RxLR_1—specific for plant parasites, oomycetes) and variants of the same motif sharing the same aa position in the protein were filtered out. For information about not-retained motifs, see column “Additional motifs identified at the same position” in Table S1.

### 4.6. Predicting 3D Protein Structures with AlphaFold

AlphaFold2-generated human OFD1 monomeric 3D model (available at Uniprot under accession O75665) was downloaded and visualized as a cartoon using Pymol version 3.1.3 [129]; aa residues within three experimentally confirmed protein-binding OFD1 motifs were additionally shown as a stick model (Figure S8a).

The structures of the OFD1 monomer and dimer were modeled using the AlphaFold3 server (https://alphafoldserver.com, Google DeepMind, London, UK) with default settings and the O75665 protein sequence as the query [130] (Figure S8b).

The default AlphaFold3 settings were also applied to predict interaction sites (Figure S9) between human OFD1 (Uniprot O75665) and one of the human proteins that bind the three experimentally confirmed motifs (e.g., GABARAP, eIF4E, and PKA, Uniprot numbers: O95166, P06730, P17612, respectively) or their respective complexes. Protein sequences for GABARAP complex contained RBCC1 (Uniprot accession: Q8TDY2); ·ATGA1 (Uniprot Q9BSB4); ATG13 (Uniprot O75143); ULK1 (Uniprot O75385). Additional proteins in the IF4E complex contained IF4G1 (Q04637) and BICC1 (Q9H694). Additional Uniprot protein sequences used to visualize human PKA complex were TBC31 (Q96DN5); PJA2 (O43164). The generated predictions were visualized in PyMOL as described previously.

Comparison of the OFD1 monomeric structures generated using various versions of AlphaFold tool indicated that both versions of the tool predict the experimentally validated eIF4E motif (aa 283–289) and LIR motif (959–964) within alpha helices, where it would typically be non-functional per ELM principles. Moreover, AF3 model of OFD1 interactions with the proteins binding the experimentally confirmed motifs (alone or in their respective complexes) fitted experimental data only for GABARAP-OFD1 interaction. For the other two motifs, AF-predicted interaction sites were localized far from the experimentally confirmed ones. Due to these inaccuracies, we have decided to use AlphaFold structures only as a visual aid.

## 5. Conclusions

OFD1 is a pleiotropic protein that plays an important role in many processes related to the centrosome/cilia and centriolar satellites. Despite many potential interactors identified in high-throughput studies, only a few have been directly confirmed with their binding sites identified. Our results suggest that OFD1 might form a scaffold for interaction with many proteins. Future research on OFD1 should focus on the regulation of OFD1 function and localization.

## Figures and Tables

**Table 1:** Number of motifs and motif types detected by ELM prediction tool in OFD1. No.—number.

No. of Motifs per Type	Before Filtering		After Filtering	
	No. of Motif Types	No. of Motifs	No. of Motif Types	No. of Motifs
1	29	29	23	23
2	13	26	2	4
3	6	18	4	12
4	6	24	5	20
5	4	20	-	-
6–10	14	110	-	-
11–20	8	115	-	-
>20	1	56	-	-
Total:	81	398	34	59

## Data Availability

The original data presented in the study are openly available in FigShare at https://doi.org/10.6084/m9.figshare.28014641.

## Supplementary Materials

The following supporting information can be downloaded at https://www.mdpi.com/article/10.3390/ijms26031167/s1.
