# Supplementary material for: Conservation of OFD1 Protein Motifs: Implications for Discovery of Novel Interactors and the OFD1 Function
Source: Int J Mol Sci. 2025 Jan 29;26(3):1167. doi: 10.3390/ijms26031167 (PMC11818881; doi:10.3390/ijms26031167)
Supplement: Supplementary file 1 [file ijms-26-01167-s001.zip › ijms-3402936-supplementary.pdf]

International Journal of
Molecular Sciences

MDPI

Conservation of OFD1 Protein Motifs: Implications for Discovery of Novel Interactors

Przemysław Jagodzik [1], Ewa Zietkiewicz [1] and Zuzanna Bukowy-Bieryllo [1,*]

Supplementary Materials

Supplementary Figures:

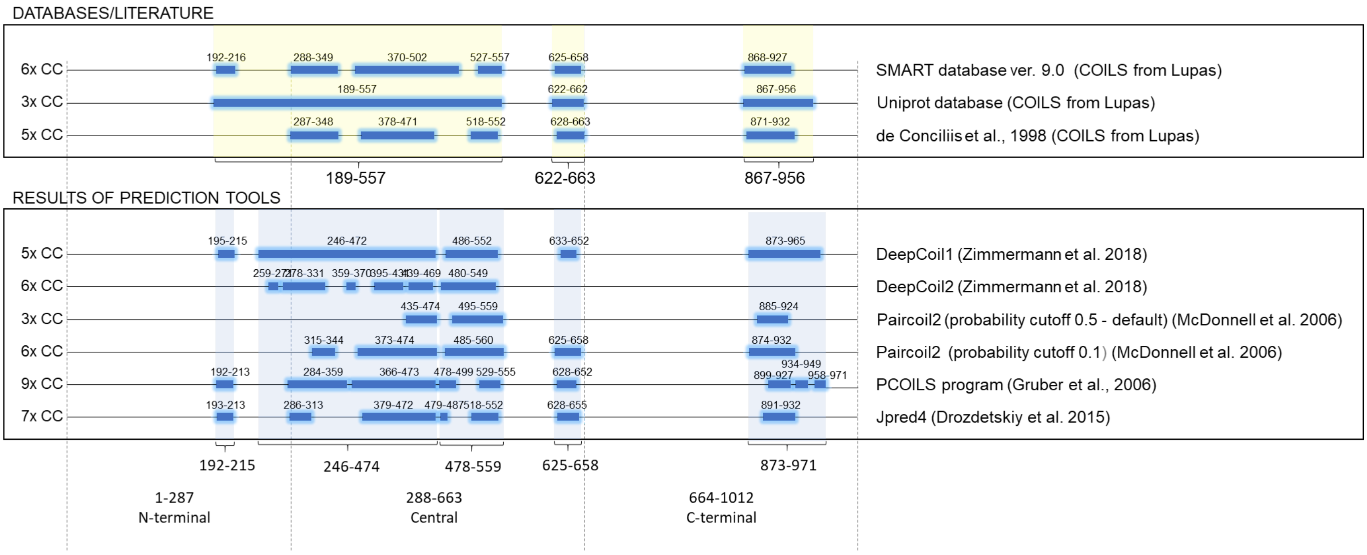

Figure S1. Comparison of CC domain architecture of OFD1 according to the various databases and prediction tools [111,113–115]

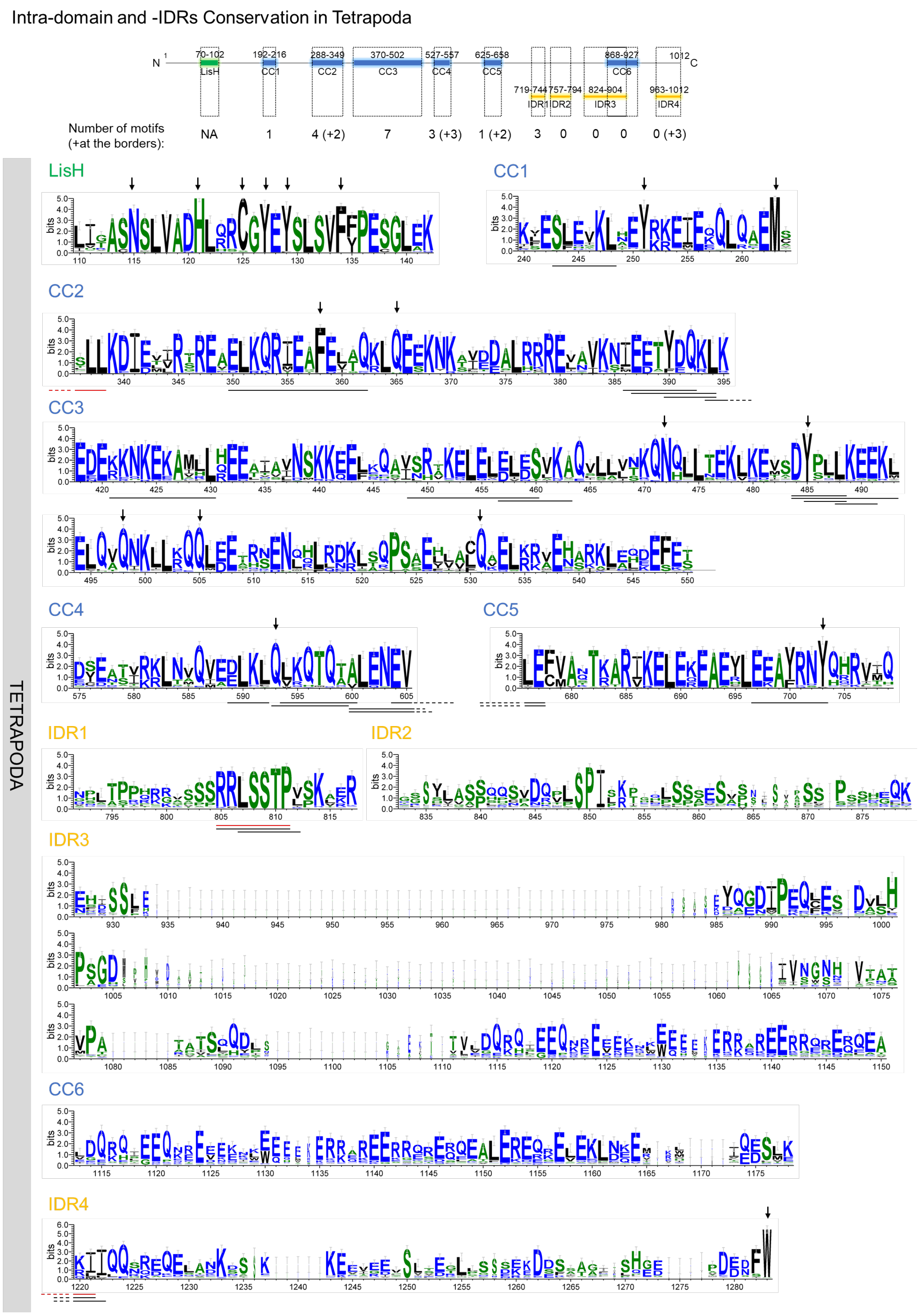

Figure S2. Graphical representation of aa position conservation of Tetrapoda OFD1 protein intra-domain/-IDRs sequences. The most highly conserved aa positions (≥ 4 bits per site) are indicated by an arrow. Highly conserved functional motifs among Tetrapoda are marked with a solid line, red line indicates experimentally confirmed motifs. The dashed line indicates the continuation of the motif beyond the area shown. A logo was created using OFD1 protein sequences from 80 species of the Tetrapoda group and aa positions are colored by hydrophobicity (blue – hydrophilic; green – neutral; hydrophobic – black). A logo was created using sequence logo generator: WebLogo [43,44].

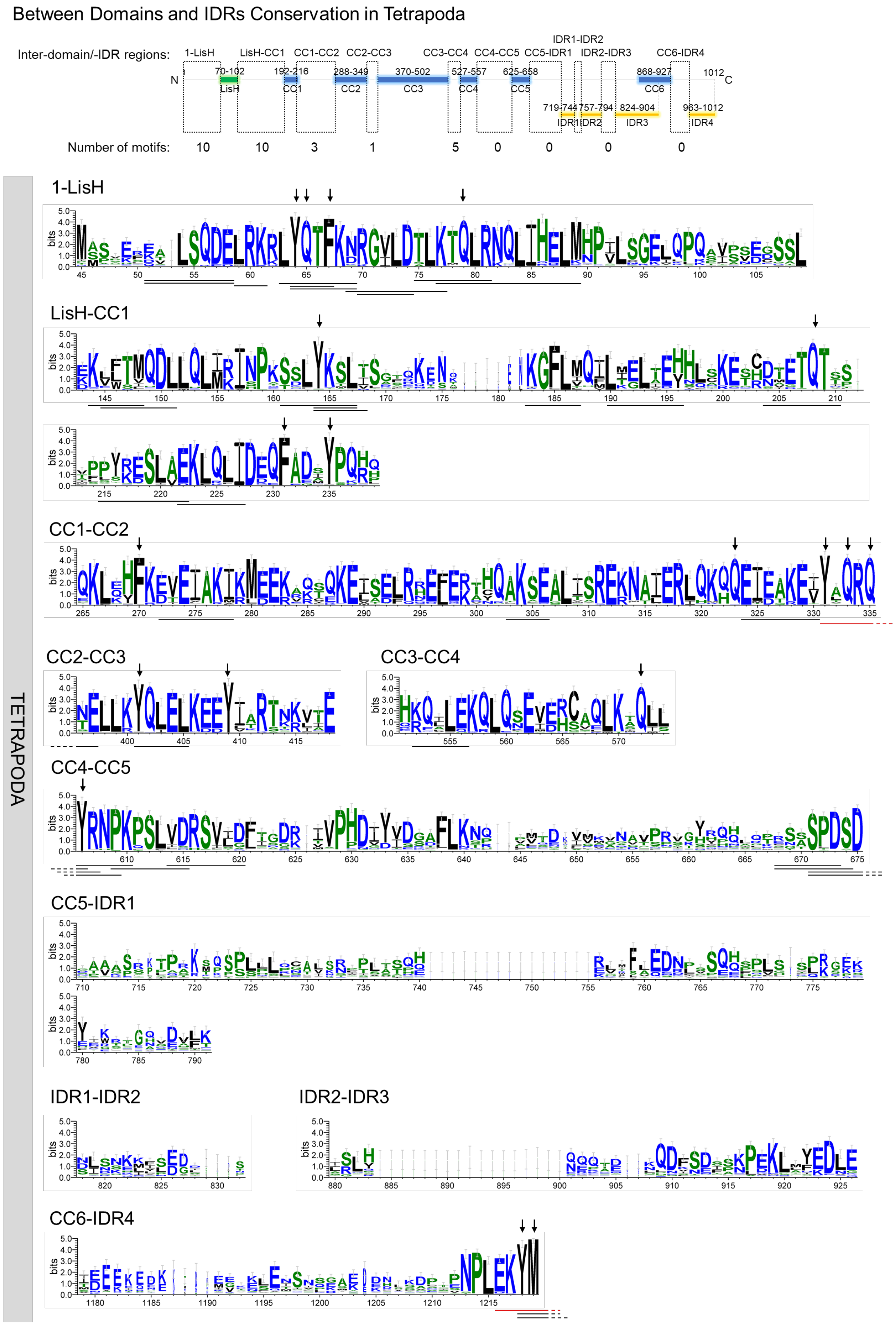

Figure S3. Graphical representation of aa position conservation of Tetrapoda OFD1 protein between domain/IDRs sequences. The most highly conserved aa positions (≥ 4 bits per site) are indicated by an arrow. Highly conserved functional motifs among Tetrapoda are marked with a solid line, red line indicates experimentally confirmed motifs. The dashed line indicates the continuation of the motif beyond the area shown. A logo was created using OFD1 protein sequences from 80 species of the Tetrapoda group and aa positions are colored by hydrophobicity (blue – hydrophilic; green – neutral; hydrophobic – black). A logo was created using sequence logo generator: WebLogo [43,44].

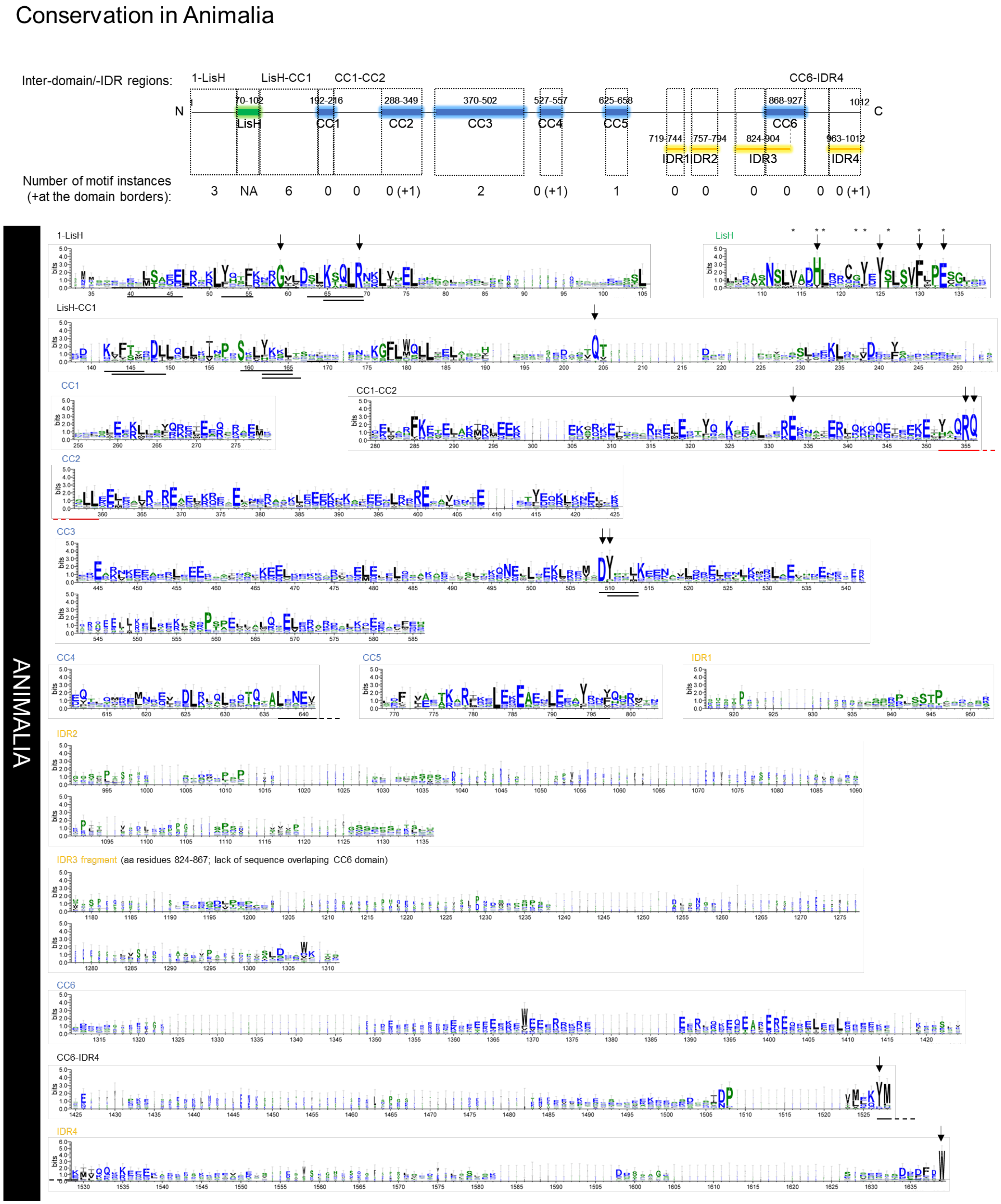

Figure S4. Graphical representation of aa position conservation in selected Animalia OFD1 protein regions. The most highly conserved aa residues (ranging from 3 to 4 bits per sit) are indicated by an arrow. Highly conserved functional motifs among Animalia are marked with a solid line. The dashed line indicates the continuation of the motif beyond the area shown. A logo was created using OFD1 protein sequences from 26 species of Animalia and aa positions are colored by hydrophobicity (blue – hydrophilic; green – neutral; hydrophobic – black). A logo was created using sequence logo generator: WebLogo [43,44]. Asterisks

indicate the most conserved aa residues among LisH domains (103 LisH entries from the Conserved Domain Database) according to [46].

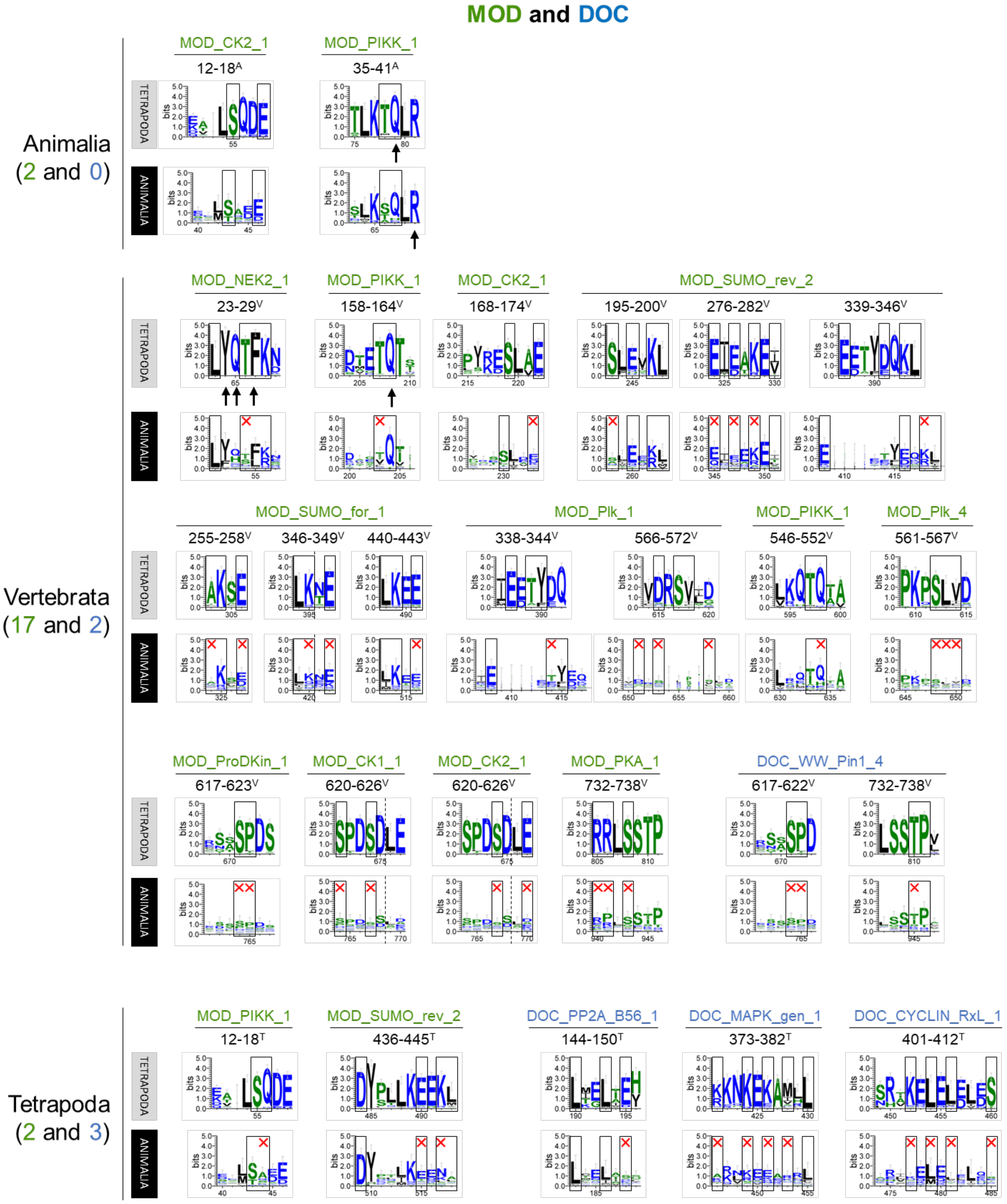

Figure S5. Sequences of the MOD and DOC motif sequences identified in human OFD1 and their conservation within different groups of animals. Motif patterns identified in human OFD1 were compared with the sequence logo graphs representing consensus sequence of Tetrapoda and Animalia alignments. Aa positions required by the motif pattern are boxed; red cross indicates aa residues not fitting to the motif pattern. The most highly conserved aa positions in the protein alignments are indicated by an arrow. Color of the aa residues reflects its hydrophobicity (blue – hydrophilic; green – neutral; hydrophobic – black). Motifs are grouped according the animal clades in which the motif was recognized as conserved.

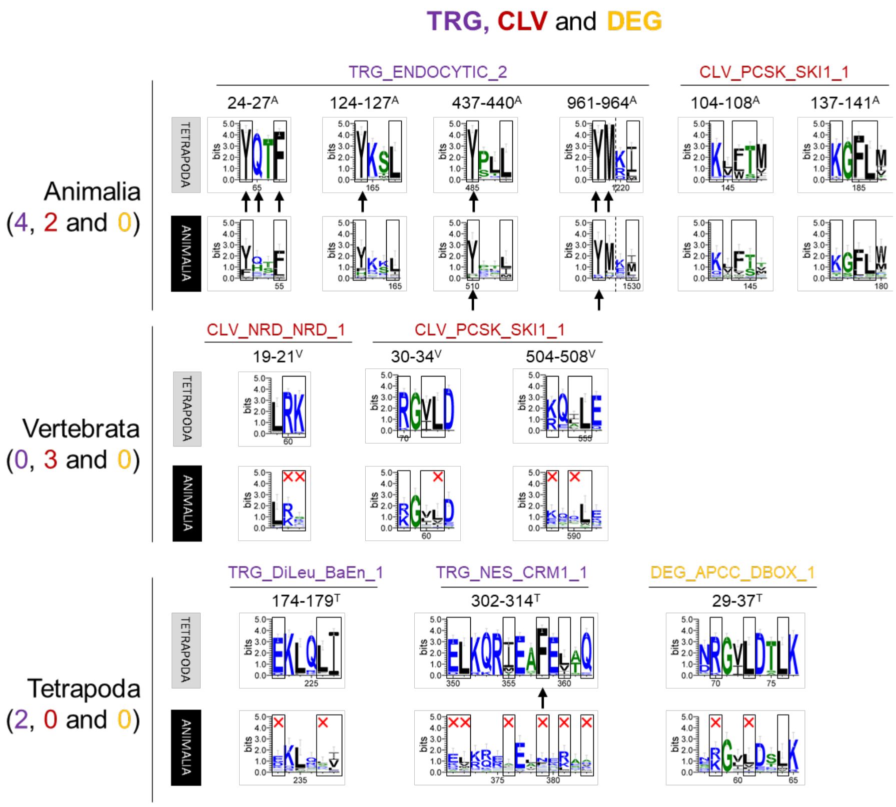

Figure S6. Sequences of the TRG, CLV and DEG motif sequences identified in human OFD1 and their conservation within different groups of animals. Motif patterns identified in human OFD1 were compared with the sequence logo graphs representing consensus sequence of Tetrapoda and Animalia alignments. Aa positions required by the motif pattern are boxed; red cross indicates aa residues not fitting to the motif pattern. The most highly conserved aa positions in the protein alignments are indicated by an arrow. Color of the aa residues reflects its hydrophobicity (blue – hydrophilic; green – neutral; hydrophobic – black). Motifs are grouped according the animal clades in which the motif was recognized as conserved.

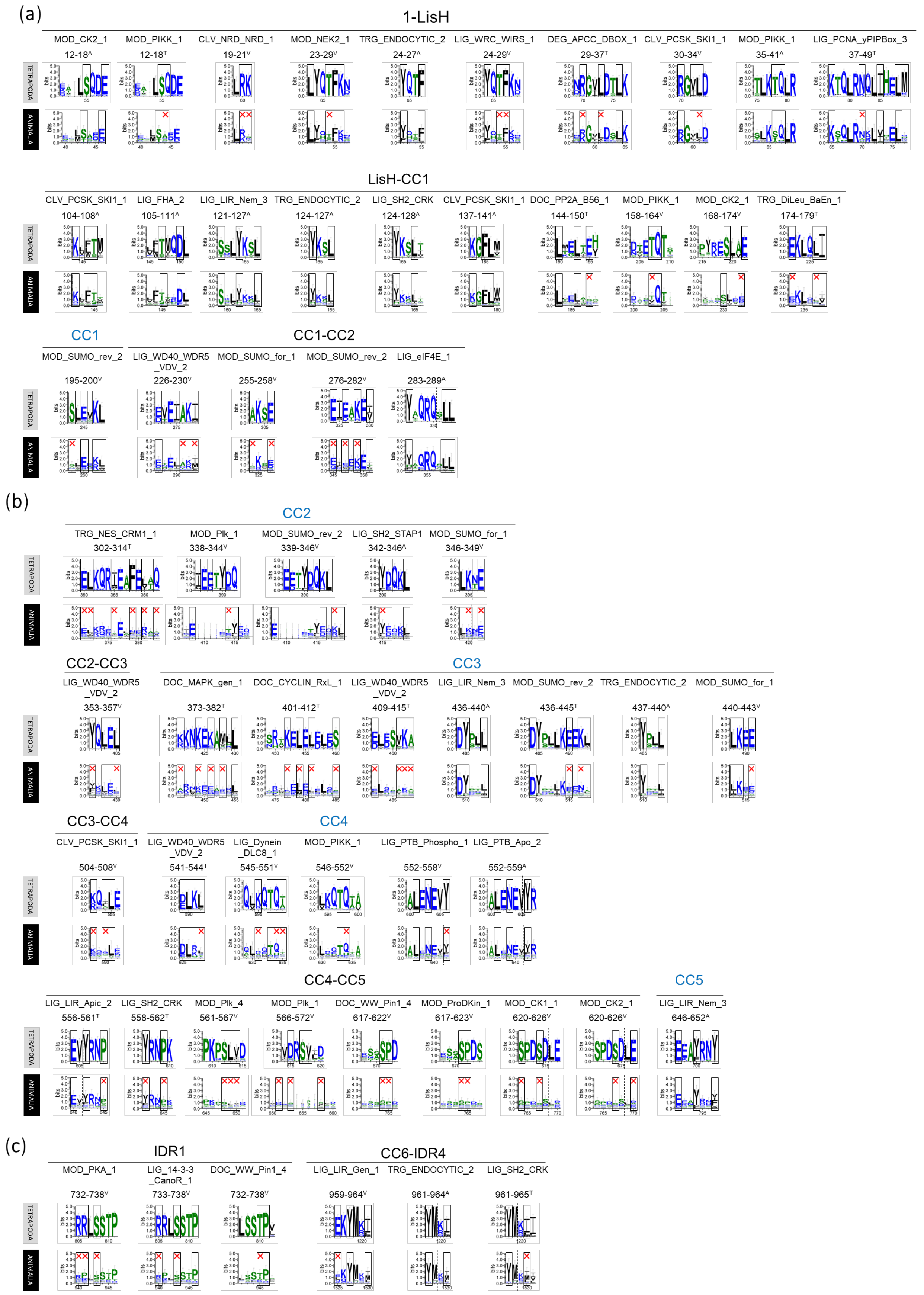

Figure S7. Distribution of all ELM motifs identified in human OFD1 regions and their conservation within different groups of animals. (**a**) N-terminal, (**b**) central and (**c**) C-terminal part of OFD1 protein. Motif patterns identified in human OFD1 were compared with the sequence logo graphs representing consensus sequence of Tetrapoda and Animalia alignments. Positions of the aa residues fitting to the motif pattern requirements are boxed. Color of the aa residues reflects its hydrophobicity (blue –

hydrophilic; green – neutral; hydrophobic – black). Index in the motif position indicates the conservation of the motif instance. A – conserved in Animalia, V – conserved among Vertebrata, T – conserved among Tetrapoda. *1-LisH* – the sequence preceding the LisH domain, *LisH-CC1* – the sequence between LisH and CC1 domain, CCx- CCy – sequence between CC domains X and Y, IDR,

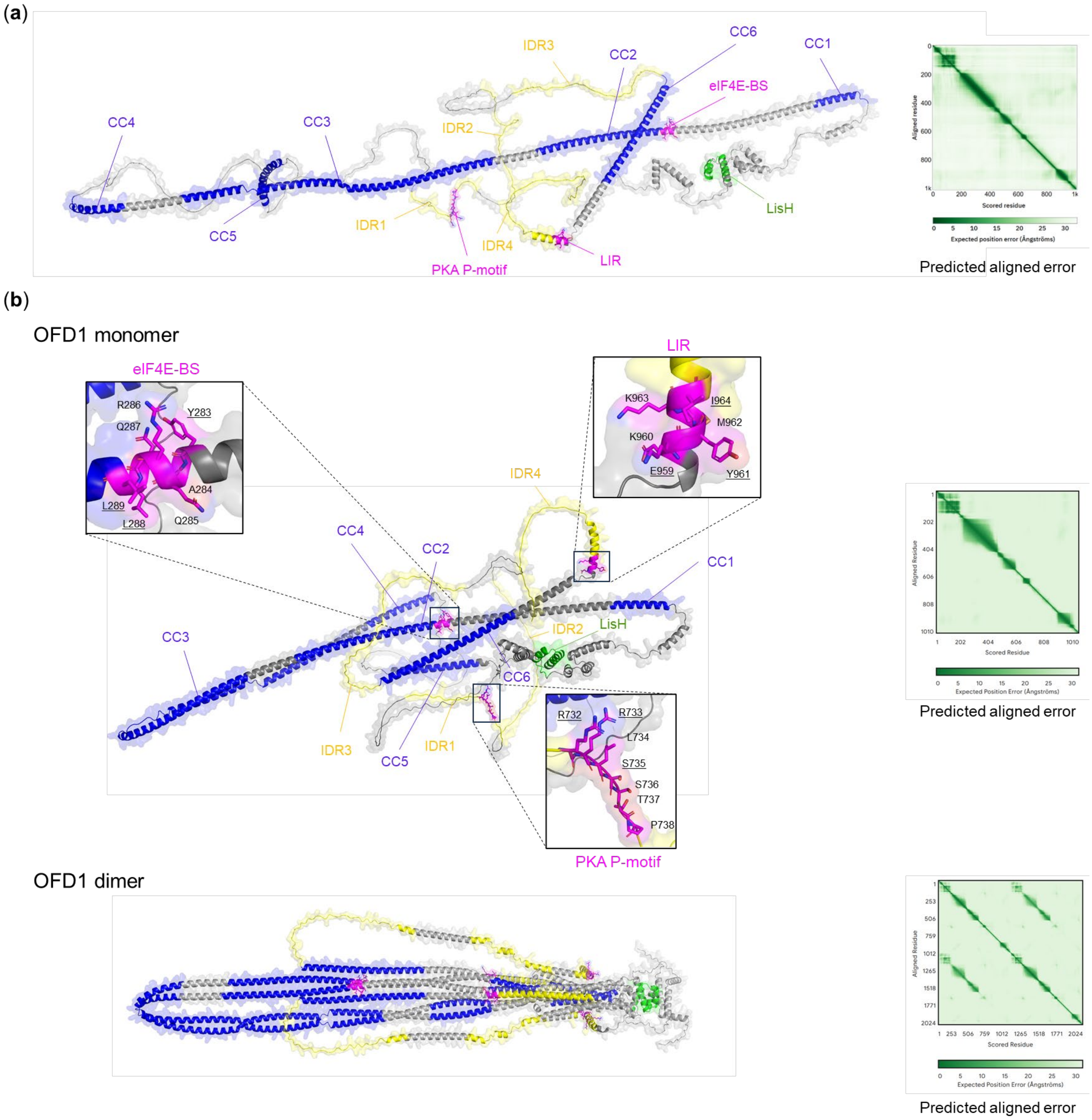

Figure S8. **AlphaFold models of OFD1 3D structure along with the predicted aligned error graphs.** (**a**) AlphaFold 2 monomeric OFD1 model from Uniprot database; (**b**) AlphaFold 3 models generated by the authors. (top) OFD1 monomer; (bottom) OFD1 dimer. OFD1 monomeric models created using different AlphaFold versions are structurally different, but bothversions of the tool predict a single CC domain in OFD1 region (aa 189–557), placing the experimentally validated eIF4E motif (aa 283–289) within a CC domain, where it would typically be non-functional per ELM principles. Structures visualized as cartoon in Pymol, experimentally confirmed protein-binding motifs are additionally shown as stick model. Coloring of the domains and motifs as in Figure 1. CC – coil-coiled domain; eIF4E-BS – eukaryotic initiation factor 4E binding site; IDR – intrinsically disordered region; LIR – LC3-interacting region, LisH – Lissencephaly type-1-like homology domain; LCR – low complexity region. PKA P-motif – protein kinase A phosphorylation motif.

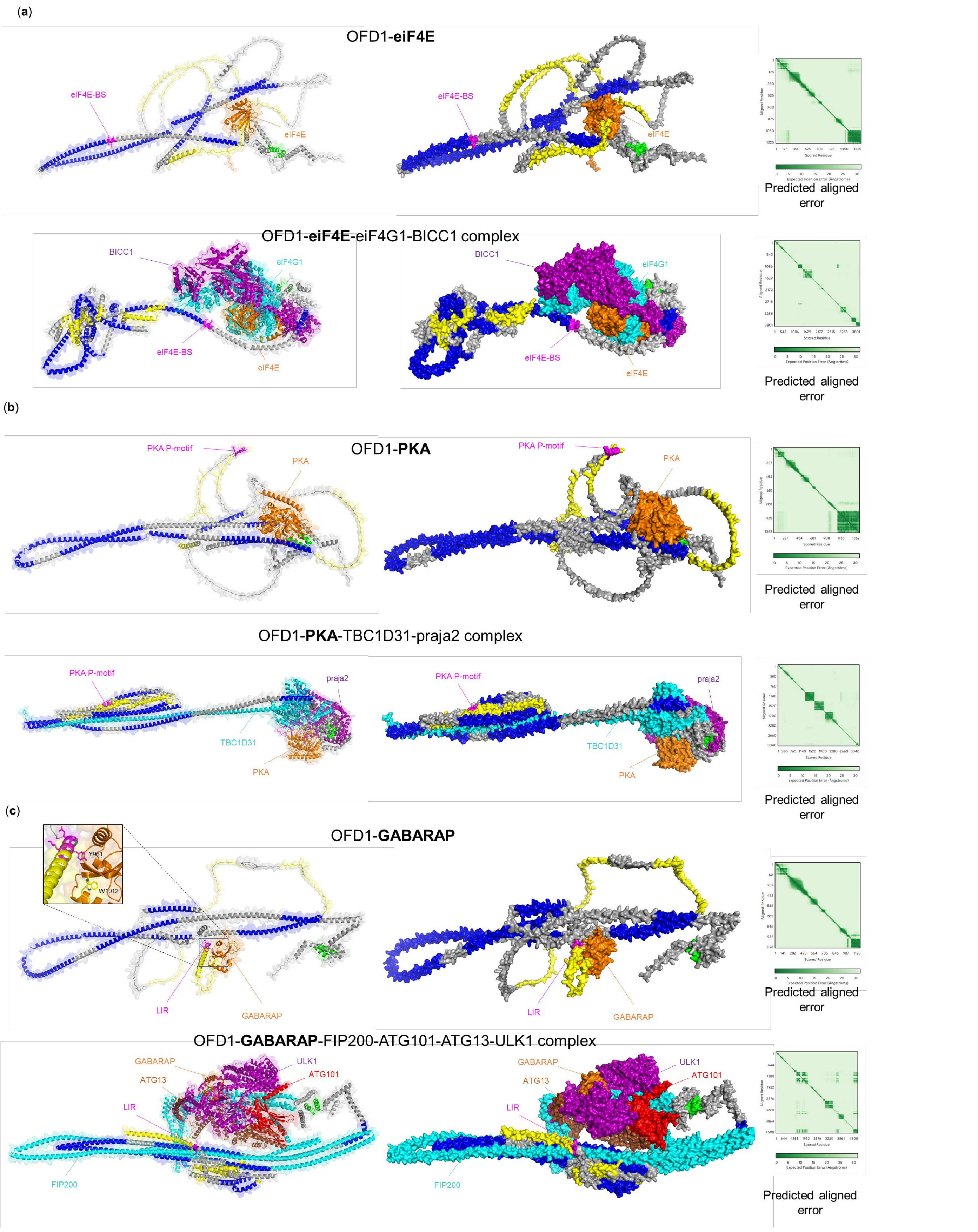

Figure S9. **Alphafold3 predictions of OFD1 3D structure interacting with its experimentally confirmed protein partners (alone or a part of their respective complexes).** Structures are predicted by AlphaFold3 and visualized by Pymol. Predicted aligned error graphs are shown on the right. Only for OFD1-GABARAP interaction, AlphaFold3 predicted the experimentally confirmed LIR

motif in close proximity of the binding protein and might interact with OFD1 protein. For the rest of the models (both two-protein or multi-protein models), AlphaFold3 predicted docking sites in OFD1 do not fit the experimental data. (**a**) Model of eIF4E interaction. Top: OFD1-eIF4E; bottom: OFD1-eIF4E-eIF4G-BICC complex. (**b**) Model of OFD1-PKA interaction. Top: OFD1-PKA; bottom: OFD1-PKA-TBC1D31-praja2 complex. (**c**) OFD1-GABARAP interaction. Top: OFD1-GABARAP; bottom: OFD1-FIP200-ATG101-ATG13-ULK1 complex. Inset shows magnification the interaction site. Respective motifs are shown in magenta and as a stick model; other structures are colored as in Figure 1. OFD1 interactors are shown in orange, purple, cyan brown or red.

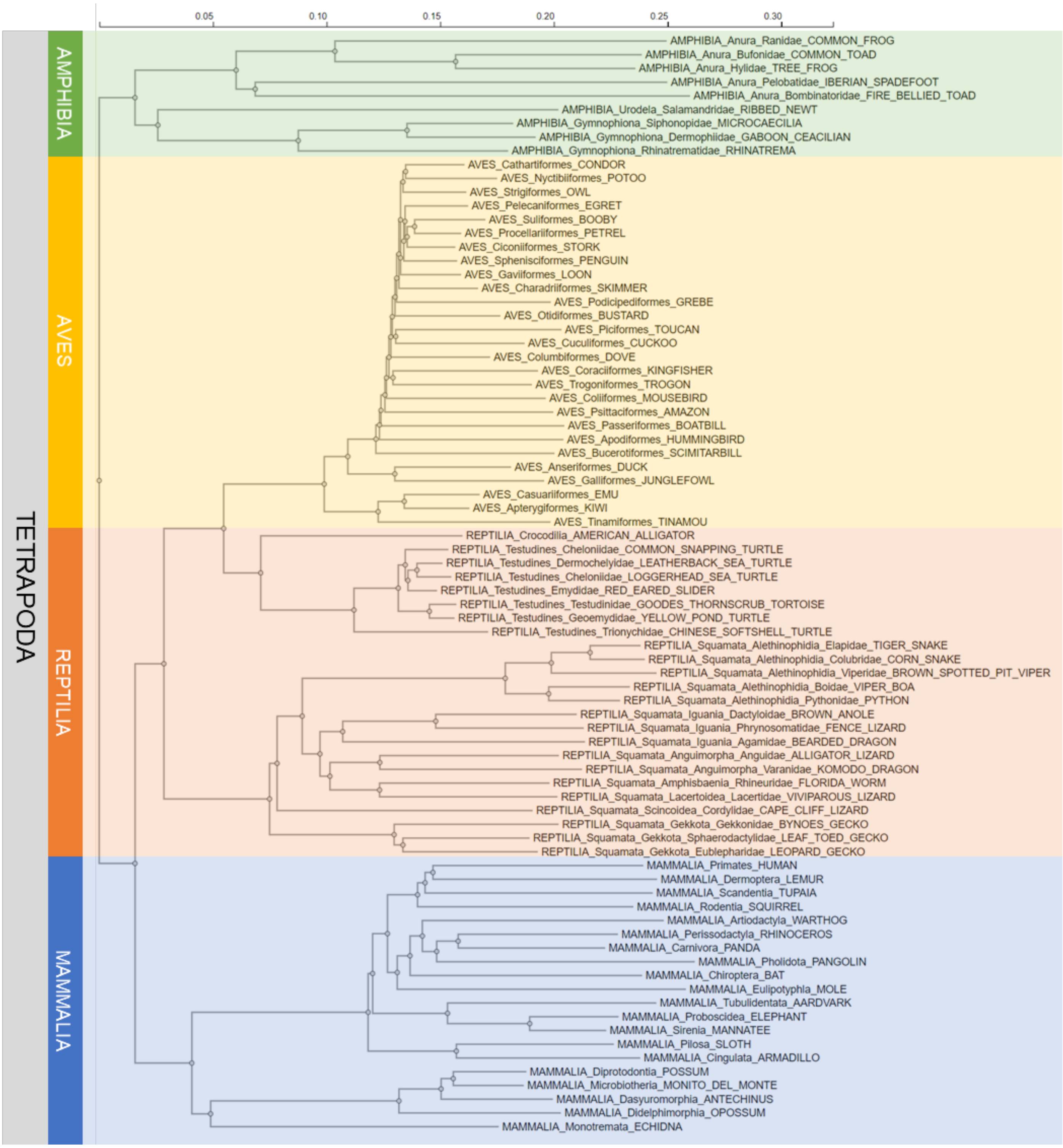

Figure S10. Phylogenetic tree of Tetrapoda OFD1 protein sequence. Animal names at the beginning of each line are highlighted depending on their phylogeny: mammals in blue, reptiles in red, birds in yellow, and amphibians in green.. The name of the

species representative (in capital letters) is preceded by the name of the family or/and order (in lowercase letters) and class of Tetrapoda (in capital letters).

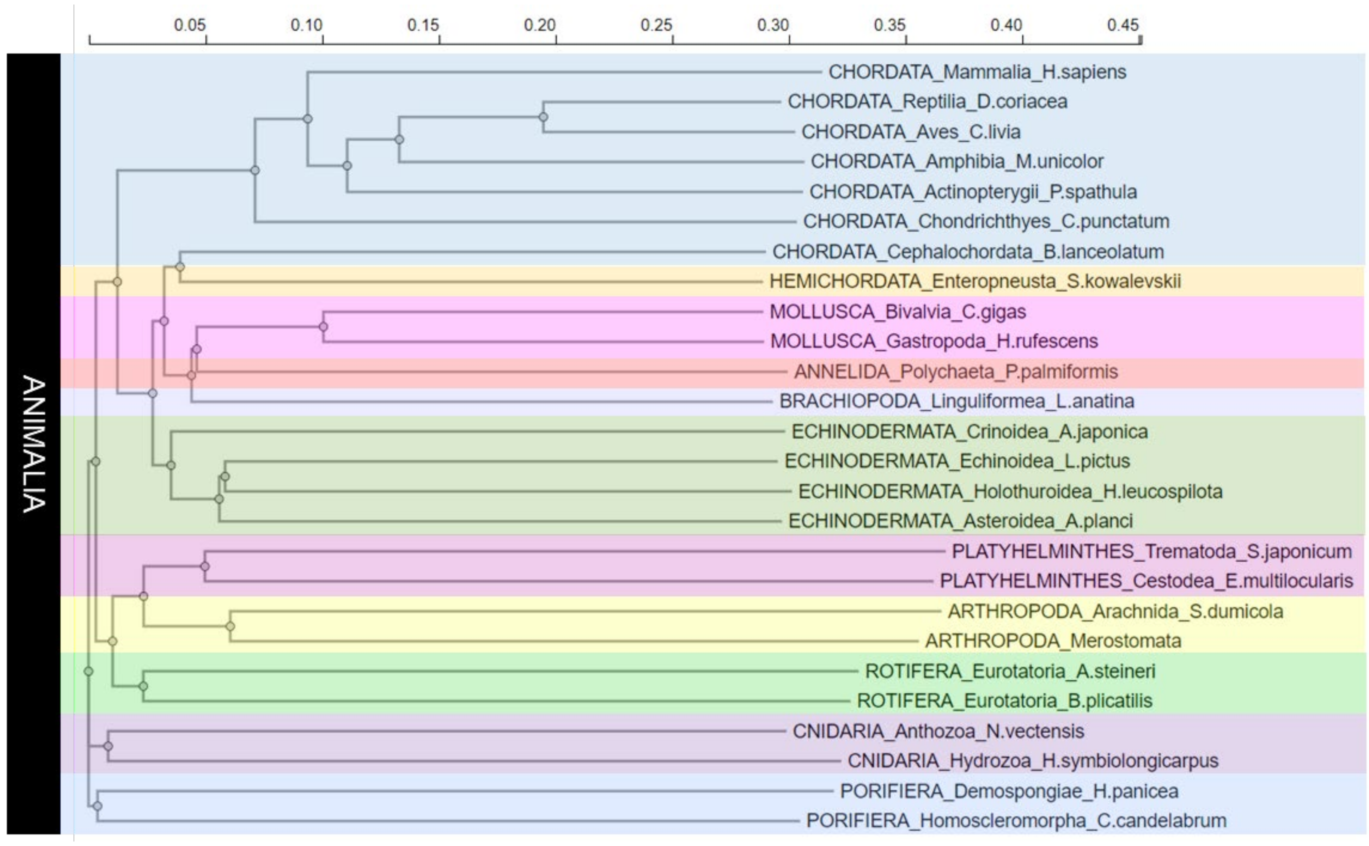

Figure S11. Phylogenetic tree of Animalia OFD1 protein sequence. Each animal phylum was indicated by different color. The name of the representative species (in lowercase) is preceded by the name of the class (in lowercase) and Animalia kingdom phylum (in capital letters).

Supplementary Tables:

Table S1. List of detected ELM motifs after filtering. Index in the motif position indicates the conservation of the motif instance. A – conserved in Animalia, V – conserved among Vertebrata, T – conserved among Tetrapoda. Positions of the aa residues fitting to the motif pattern requirements are underlined.

Elm Name	Elm description	OFD1 protein position	Motif sequence	Additional motifs identified at the same position
CLV_NRD_NRD_1	N-Arg dibasic convertase (NRD/Nardilysin) cleavage site (X-|-R-K or R-|-R-X).	19-21^{V}	LRK	-
CLV_PCSK_SKI1_1	Subtilisin/kexin isozyme-1 (SKI1) cleavage site ([RK]-X-[hydrophobic]-[LTKF]-|-X)	30-34^{V}<br>104-108^{A}<br>137-141^{A}<br>504-508^{V}	RGILD<br>KVFTM<br>KGFLM<br>RQALH	-<br>-<br>-<br>-
DEG_APCC_DBOX_1	An RxxL-based motif that binds to the Cdh1 and Cdc20 components of APC/C	29-37^{T}	DRGILDTLK	-
DOC_CYCLIN_RxL_1	Both fungal and mammalian S-phase Cyclin/CDK	401-412^{T}	NRVKELELELES	-

	complexes recognize specific RxL docking motifs in their target proteins.			
DOC_MAPK_gen_1	MAPK interacting molecules (e.g. MAPKKs, substrates, phosphatases) carry docking motif that help to regulate specific interaction in the MAPK cascade. The classic motif approximates (R/K)xxxxx where is a hydrophobic residue.	373-382[T]	RKNKEKAVHL	DOC_MAPK_Gen_1 (shorter: KNKEKAVHL, 374-382[T])
DOC_PP2A_B56_1	Docking site required for the regulatory subunit B56 of PP2A for protein dephosphorylation.	144-150[T]	LKELAEY	
DOC_WW_Pin1_4	The Class IV WW domain interaction motif is recognised primarily by the Pin1 phosphorylation-dependent prolyl isomerase.	617-622[V]<br>732-738[V]	EGSSPD<br>LSSTPL	-
LIG_14-3-3_CanoR_1	Canonical Arg-containing phospho-motif mediating a strong interaction with 14-3-3 proteins.	733-738[V]	RRLSSTP	LIG_14-3-3_CanoR_1 (shorter: RLSSTP, 734-738[V])
LIG_Dynein_DLC8_1	The [KR]xTQT motif interacts with the common target-accepting grooves of 8kDa Dynein Light Chain dimer.	545-551[V]	QLKQTQT	-
LIG_eIF4E_1	Motif binding to the dorsal surface of eIF4E.	283-289[A]	YAQRQLL	-
LIG_FHA_2	Phosphothreonine motif binding a subset of FHA domains that have a preference for an acidic aa at the pT+3 position.	105-111[A]	VFTMQDL	-
LIG_LIR_Apic_2	Apicomplexa specific variant of the canonical LIR motif that binds to Atg8 protein family members to mediate processes involved in autophagy.	556-561[T]	EVYCNP	-
LIG_LIR_Gen_1	Canonical LIR motif that binds to Atg8/LC3 protein family members to mediate processes involved in autophagy.	959-964[V]	EKYMKI	LIG_LIR_Nem_3 (EKYMKI, 959-964[V]) and LIG_LIR_Gen_1 (longer: EKYMKII, 959-965[V])
LIG_LIR_Nem_3	Nematode-specific variant of the canonical LIR motif that binds to Atg8 protein family members to mediate	121-127[A]	SSLYKSL	

	processes involved in autophagy.	436-440^{A}<br>646-652^{A}	DYSLL<br>EKAFRSY	-
LIG_PCNA_yPIPBox_3	The PCNA binding motifs include the PIP Box, PIP degron and the APIM motif, and are found in proteins involved in DNA replication, repair, methylation and cell cycle control. This is the variant for the yeast PIPbox	37-49^{T}	KTQLRNQLI-HELM	-
LIG_PTB_Apo_2	These phosphorylation-independent motifs bind to Dab-like PTB domains. Binding is not driven by contacts at the 0 or FY position, but instead is dependent upon the large number of hydrophobic and hydrogen bond contacts between motif and domain.	552-559^{A}	ALENEVYC	-
LIG_PTB_Phospho_1	This phosphorylation-dependent motif binds to Shc-like and IRS-like PTB domains. The pTyr is positioned within a highly basic-charged anchoring pocket. A hydrophobic residue -5 (compared to pY) increases the affinity of the interaction.	552-558^{V}	ALENEVY	
LIG_SH2_CRK	CRK family SH2 domain binding motif	124-128^{A}<br>558-562^{T}<br>961-965^{T}	YKSLV<br>YCNPK<br>YMKII	-
LIG_SH2_STAP1	STAP1 Src Homology 2 (SH2) domain Class 2 binding motif	342-346^{A}	YDRKL	-
LIG_WD40_WDR5_VDV_2	Fungi-specific variant of the WDR5-binding motif that binds to a cleft between blades 5 and 6 of the WD40 repeat domain of WDR5, opposite of the Win motif-binding site, to mediate assembly of histone modification complexes.	224-230^{V}	DTEIAKI	LIG_WD40_WDR5_VDV_2 (less conserved: EIAKI, 226-230^{T})
		353-357^{V}	YQLEL	
		409-415^{T}	ELESVKA	LIG_WD40_WDR5_VDV_2 (two shorter: ESVKA,411-415^{T};SVKA, 412-415^{T})
		541-544^{T}	DLKL	
LIG_WRC_WIRS_1	WRC interacting receptor sequence (WIRS) is a highly	24-29^{V}	YQTFKD	

	conserved and widespread interaction motif that is employed by diverse membrane proteins to recruit the WRC to initiate the dynamic rearrangements of the actin cytoskeleton.			
MOD_CK1_1	CK1 phosphorylation site	620-626[V]	SPDSDLE	
MOD_CK2_1	Casein kinase 2 (CK2) phosphorylation site	12-18[A]	DVLSQDE	
		168-174[V]	NRDSLAE	
		620-626[V]	SPDSDLE	
MOD_NEK2_1	strict version of the motif targeted by NEK2 for phosphorylation	23-29[V]	LYQTFKD	MOD_NEK2_2 (more tolerant: LYQTFKD, 23-29[V])
MOD_PIKK_1	(ST)Q motif which is phosphorylated by PIKK family members.	12-18[T]	DVLSQDE	
		35-41A[A]	TLKTQLR	
		158-164[V]	NMETQTS	
		546-552[V]	LKQTQTA	
MOD_PKA_1	Main preference for PKA-type AGC kinase phosphorylation.	732-738[V]	RRLSSTP	MOD_PKA_2 (more tolerant: RRLSSTP, 732-738[V])
MOD_Plk_1	Ser/Thr residue phosphorylated by the Plk1 kinase	338-344[V]	FEETYDR	
		566-572[V]	IDRSVNG	
MOD_Plk_4	Ser/Thr residue phosphorylated by Plk4	561-567[A]	PKQSVID	
MOD_ProDKin_1	Proline-Directed Kinase (e.g. MAPK) phosphorylation site in higher eukaryotes.	617-623[V]	EGSSPDS	
MOD_SUMO_for_1	Motif recognised for modification by SUMO-1	255-258[V]	AKSE	
		345-349[V]	LKNE	
		440-443[V]	LKEE	
MOD_SUMO_rev_2	Inverted version of SUMOylation motif recognized for modification by SUMO-1	195-200[V]	SLEIKL	MOD_SUMO_rev_2 (less conserved: ESLEIKL, 194-200[T])
		276-282[V]	EIETKEI	
		339-346[V]	EETYDRKL	MOD_SUMO_rev_2 (shorter: ETYDRKL, 340-346[V])
		436-445[T]	DYSLLKEEKL	
TRG_DiLeu_BaEn_1	Classical adaptin sigma subunit-binding acidic dileucine motifs sorting in Endosomal-Basolateral trafficking	174-179[T]	EKLQLI	
TRG_ENDOCYTIC_2	Tyrosine-based sorting signal responsible for the interaction with mu subunit of AP (Adaptor Protein) complex	24-27[A]	YQTF	
		124-127[A]	YKSL	

		437-440[A]	YSLL
		961-964[A]	YMKI
TRG_NES_CRM1_1	Many proteins re-exported from the nucleus contain an amphipathic often Leucine-rich nuclear export signal (NES) binding to the CRM1 exportin protein	302-314[T]	ELKQRVEA-FELNQ

Table S2. LIG motifs in OFD1 and their interactors. Index in the motif position indicates the conservation of the motif instance. A-conserved in Animalia, V –conserved among Vertebrata, T –conserved among Tetrapoda.

**Motif Name**	**Motif position in OFD1**	**Binding Partner Name**	**Main function**	**Other functions**	**Main localization**	**Interaction with OFD1**
LIG_14-3-3_CanoR_1	733-738^{V}	14-3-3 proteins	Signal transduction	Ciliary function; Satelite function; Centrosome function; Cell cycle; DNA repair; Transcription; Proteasomal degradation; Autophagy; Cell metabolism; Intracelullar trafficking; Apoptosis; Stress response; Malignant transformation	Cytoplasm/Nucleus	-
LIG_Dynein_DLC8_1	545-551^{V}	Dynein Light Chain 8 (DYNLL1)	Intracellular transport [57]	Ciliary function; Centrosome function; Cell cycle; Transcription	Cytoplasm	**indirect** (proximity labeling: [40,47], direct (coIP: [48–50])
LIG_eIF4E_1	283-289^{A}	Eukaryotic translation initiation factor 4E (eIF4E)	mRNA translation initiation [11]	-	Cytoplasm	**motif confirmed** (mutagenesis & coIP: [11], indirect (proximity labeling techniques: [40])
LIG_FHA_2	105-111^{A}	Forkhead-associated (FHA) domain-containing proteins	Signal transduction	Centrosome function; Cell cycle; DNA repair; Chromatin remodelling; Transcription	Nucleus	
LIG_LIR_Apic_2	556-561^{T}	Autophagy-related protein 8 (Atg8) family	Autophagy		Cytoplasm	
LIG_LIR_Gen_1	959-964^{V}	Autophagy-related protein 8 (Atg8) family	Autophagy [12]		Cytoplasm	**motif confirmed** (mutagenesis & coIP: [12])
LIG_LIR_Nem_3	121-127^{A} 436-440^{A} 646-652^{A}	Autophagy-related protein 8 (Atg8) family	Autophagy		Cytoplasm	
LIG_PCNA_yPIPBox_3	37-49^{T}	Proliferating Cell Nuclear Antigen (PCNA)	DNA replication/repair [56]	Centrosome function; Cell cycle; Chromatin remodelling; Transcription	Nucleus	-
LIG_PTB_Apo_2	552-559^{A}	Dab-like Phosphotyrosine Binding (PTB) domain-containing proteins	Signal transduction	Cell adhesion/ migration	Cytoplasm	-
LIG_PTB_Phospho_1	552-558^{V}	Shc-like and Insulin Receptor Substrate (IRS) PTB domain-containing proteins	Signal transduction		Cytoplasm/Nucleus	-
LIG_SH2_CRK	124-128^{A} 558-562^{T} 961-965^{T}	CRK (CT10 Regulator of Kinase) Adaptor Protein family	Signal transduction	Ciliary function; Cytoskeleton dynamics	Cytoplasm/ Ciliary basal body	-

LIG_SH2_STAP1	342-346[A]	Signal Transducing Adaptor Protein 1 (STAP1)	Signal transduction	Centrosome function; Cell cycle; Transcription	Cytoplasm/ Plasma membrane	-
LIG_WD40_WDR5_VDV_2	224-230[V] 353-357[V] 409-415[T] 541-544[T]	WD Repeat Domain 5 (WDR5)	Histone modification [55]	Ciliary function; Centrosome function; Cell cycle; DNA repair; Chromatin remodelling; Transcription	Nucleus	-
LIG_WRC_WIRS_1	24-29[V]	WAVE Regulatory Complex (WRC)	Actin cytoskeleton rearrangements [54]		Plasma Membrane	-

Table S3. MOD motifs in OFD1 and their interactors. Index in the motif position indicates the conservation of the motif instance. A- conserved in Animalia, V –conserved among Vertebrata, T –conserved among Tetrapoda.

Motif Name	Motif position in OFD1	Binding Partner Name	Main function	Other functions	Main localization	Interaction with OFD1
MOD_CK1_1	620-626^{V}	Casein Kinase 1 (CK1)	Phosphorylation	Ciliary function; Centrosome function; Cell cycle; DNA repair; Proteasomal degradation; Autophagy; Cell metabolism; Cytoskeleton dynamics; Signal transduction; Apoptosis; Circadian rhytms; Cell-cell adhesion	Cytoplasm/Nucleus	-
MOD_CK2_1	12-18^{A}<br>168-174^{V}<br>620-626^{V}	Casein kinase 2 (CK2)	Phosphorylation	Ciliary function; Centrosome function; Cell cycle; DNA repair; Chromatin remodelling; Transcription; Translation; Proteasomal degradation; Cell metabolism; Intracelullar trafficking; Signal transduction; Apoptosis	Cytoplasm/Nucleus	-
MOD_NEK2_1	23-29^{V}	Never in Mitosis A-related Kinase 2 (Nek2)	Cell cycle regulation	Ciliary function; Centrosome function; Proteasomal degradation; Autophagy; Cytoskeleton dynamics; Signal transduction	Centrosome	**-**
MOD_PIKK_1	12-18^{T}<br>35-41A^{A}<br>158-164^{V}<br>546-552^{V}	Phosphoinositide 3-Kinase-related Kinase (PIKK) family	DNA damage response	Ciliary function; Centrosome function; Cell cycle; Chromatin remodelling; Transcription; Cell growth regulation	Nucleus/ Cilia	**-**
MOD_PKA_1	732-738^{V}	Protein Kinase A (PKA)	Phosphorylation	Ciliary function; Transcription; Cell metabolism; Signal transduction; Cell growth; Apoptosis; Stress responses	Cytoplasm/Nucleus	**motif confirmed** (mutagenesis & coIP [8])
MOD_Plk_1	338-344^{V}<br>566-572^{V}	Polo-like Kinase 1 (Plk1)	Cell cycle regulation	Ciliary function; Centrosome function; DNA repair; Proteasomal degradation; Autophagy	Cytoplasm/Nucleus/ Mitotic spindle	**direct** (Y2H: [51], indirect (proximity labeling techniques [40,51])
MOD_Plk_4	561-567^{A}	Polo-like Kinase 4 (Plk4)	Cell cycle regulation	Centrosome function; DNA repair; Autophagy	Cytoplasm/ Centrosome	**indirect** (proximity labeling techniques [52])
MOD_ProD-Kin_1	617-623^{V}	Proline-Directed Kinases	Phosphorylation	Ciliary function; Satelite function; Centrosome function; DNA repair; Chromatin remodelling; Transcription; Autophagy; Signal transduction	Cytoplasm/Nucleus	-
MOD_SUMO_for_1	255-258^{V}<br>345-349^{V}	Small Ubiquitin-like Modifier 1 (SUMO-1)	Post-translational modification	Ciliary function; Centrosome function; Cell cycle; DNA repair; Chromatin	Nucleus/Cytoplasm	-

	440-443[V]			remodelling; Transcription; Translation; Proteasomal degradation		
MOD_SUMO_rev_2	195-200[V]<br>276-282[V]<br>339-346[V]<br>436-445[T]	Small Ubiquitin-like Modifier 1 (SUMO-1)	Post-translational modification	Ciliary function; Centrosome function; Cell cycle; DNA repair; Chromatin remodelling; Transcription; Translation; Proteasomal degradation	Nucleus/Cytoplasm	-

Table S4. DOC motif-binding interactors of OFD1. A- motif instances conserved in Animalia, V – motif instances conserved among Vertebrata, T – motif instances conserved among Tetrapoda.

Motif Name	Motif position in OFD1	Binding Partner Name	Main function	Other functions	Main localization	Interaction with OFD1
DOC_CYCLIN_RxL_1	401-412[T]	Cyclins and Cyclin-dependent kinases (CDKs)	Cell cycle regulation	Centrosome function; DNA repair; Transcription	Cytoplasm/Nucleus	**indirect** (proximity labeling techniques [40,53])
DOC_MAPK_gen_1	373-382[T]	Mitogen-Activated Protein Kinase (MAPK)	Signal transduction	Ciliary function; Satelite function; Centrosome function; Cell cycle; DNA repair; Chromatin remodelling; Transcription; Autophagy	Cytoplasm/Nucleus	-
DOC_PP2A_B56_1	144-150[T]	Protein Phosphatase 2A (PP2A)	Protein dephosphorylation	Centrosome function; Cell cycle; Proteasomal degradation; Autophagy; Cytoskeleton dynamics; Signal transduction	Cytoplasm/Nucleus	-
DOC_WW_Pin1_4	617-622[V]<br>732-738[V]	Peptidyl-prolyl cis-trans isomerase NIMA-interacting 1 (Pin1)	Prolyl isomerization	Centrosome function; Cell cycle; DNA repair; Chromatin remodelling; Transcription; Translation; Proteasomal degradation; Autophagy; Signal transduction	Cytoplasm/Nucleus	-

Table S5. TRG, CLV and DEG motif-binding interactors of OFD1. A- motif instances conserved in Animalia, V – motif instances conserved among Vertebrata, T – motif instances conserved among Tetrapoda.

Motif Name	Motif position in OFD1	Binding Partner Name	Main function	Other functions	Main localization	Interaction with OFD1
TRG_DiLeu_BaEn_1	174-179^{T}	Sigma subunit of AP-1, AP-2, and AP-3 complexes	Vesicular trafficking	-	Endosomal/Basolateral Membrane	-
TRG_ENDOCYTIC_2	24-27^{A}<br>124-127^{A}<br>437-440^{A}<br>961-964^{A}	Mu subunit of AP-2 complex	Endocytosis	-	Plasma Membrane	-
TRG_NES_CRM1_1	302-314^{T}	Chromosome Region Maintenance 1 (CRM1)	Nuclear export		Nucleus	
CLV_NRD_NRD_1	19-21^{V}	Nardilysin (NRD)	Proteolytic processing	Ciliary function; Intracelullar trafficking	Cytoplasm/Mitochondria	-
CLV_PCSK_SKI1_1	30-34^{V}<br>104-108^{A}<br>137-141^{A}<br>504-508^{V}	Subtilisin/kexin isozyme-1 (SKI1)	Proteolytic processing	Intracelullar trafficking	Golgi apparatus	-
DEG_APCC_DBOX_1	29-37^{T}	Anaphase-Promoting Complex/Cyclosome (APC/C)	Protein degradation	Centrosome function; Cell cycle; Autophagy	Cytoplasm/Nucleus	-
